# Neural and Endocrinal Pathobiochemistry of Vitiligo: Comparative Study for a Hypothesized Mechanism

**DOI:** 10.3389/fendo.2018.00197

**Published:** 2018-04-25

**Authors:** Mohamed-I. Kotb El-Sayed, Ahmed A. Abd El-Ghany, Refaat R. Mohamed

**Affiliations:** ^1^Biochemistry and Molecular Biology Department, Faculty of Pharmacy, Helwan University, Ain Helwan, Cairo, Egypt; ^2^Biochemistry Department, Faculty of Pharmacy, Al-Azhar University, Assuit Branch, Assuit, Egypt; ^3^Dermatology, Andrology and STDs Department, Faculty of Medicine, Al-Azhar University, Assuit Branch, Assuit, Egypt

**Keywords:** vitiligo, thyroid hormones, autoimmune, estrogen, catecholamine, prolactin, melatonin

## Abstract

The etiology of vitiligo is still unclear. The aim is to investigate a neural and hormonal etio-pathology of vitiligo. Sixty acrofacial vitiligo patients were divided into two subgroups as active vitiligo patients group (AVPs; *n* = 35) and stable vitiligo patients group (SVP; *n* = 25). Forty healthy subjects without any systemic or dermatological disease were used as controls. Blood samples were collected, and the samples were used for measurement of free triiodothyronine (fT_3_), free thyroxine (fT_4_), thyroid-stimulating hormone (TSH), adrenocorticotrophic hormone (ACTH), cortisol, estrogen, testosterone, melatonin, and prolactin levels by ELISA, while norepinephrine (NE), epinephrine (Epi), dopamine (DA), homo-vanillic acid (HVA), serotonin, and 5-hydroxyindoleacetic acid (5-HIAA) by high-pressure liquid chromatography. The current results showed a significant increase in plasma levels of Epi, NE, DA, HVA, serotonin, 5-HIAA, melatonin, and in serum level of TSH and prolactin either in SVP or AVP groups than the control group and in AVP than SVP group. The serum levels of fT_3_ and fT_4_ were significantly decreased either in SVP or AVP groups than the control group. A significant increase in estradiol levels was observed in females within AVP than females in either SVP or control groups. There was a significant increase in serum level of cortisol in AVP than either SVP or control group. There was a significant decrease in serum level of ACTH in either AVP or SVP than control and in AVP than SVP group. In conclusion, there are some neural and endocrine markers that play a pivotal role in pathogenesis and/or consequences of vitiligo. The abnormally disturbed levels of theses markers lead to melanocyte destruction and/or depigmentation.

## Introduction

Vitiligo (also known as leukoderma) is a chronic skin disease characterized by skin depigmentation disorder with well-defined asymptomatic white patches of different size and shape from skin. It is considered to have an autoimmune basis responsible for melanocytes destruction (1). Patients with stable diseases have the stability of depigmented patches while those with active diseases have a speedy appearance of new lesions (2) and acrofacial vitiligo means the depigmentation in the face and distal extremities (3).

Several hypotheses have been proposed to explain the causes of melanocyte dysfunction. These include: an autoimmune mechanism (4), an altered tetrahydrobiopterin (BH_4_) homeostasis (5) psychological stressor (6) a neural hypothesis (7), hormonal changes and stressful hypothesis (8), and an imbalance between oxidative stress and antioxidant defense effect that affect melanin production (9) singularly or in combination.

The neural and stressful hypothesis of vitiligo etiology is supported by different findings. The level of catecholamines and their metabolites are released as a consequence of emotional and/or stressful events is considered as being strictly related to vitiligo (7). Moreover, skin cells also possess fully functional serotonin and melatoninergic systems. Melatonin is a product of the multistep conversion of l-tryptophan to serotonin and then melatonin. Melatonin synthesis is not restricted to the pineal gland, but was detected in several organs, including skin with the activity of a neurotransmitter, antioxidant, modulation of melanogenesis, melanoma growth control, and global regulator of the circadian clock (10, 11).

Prolactin is a hormone secreted by the anterior pituitary gland and seems to have additional functions in skin biology and hair follicle growth as well. It is a growth factor for lymphocytes with the potential to stimulate immune responses at many levels (12).

It has been provided an evidence for the presence of oxidative stress and impairment in the antioxidant system in the skin and in blood cells of vitiligo patients (VP) that results in a free radical-mediated damage in melanocyte (9). It is noted that the generation of hydrogen peroxide (H_2_O_2_) by estrogens can contribute to DNA damage, so it has been suggested that estrogens may be involved in the depigmentation process of vitiligo (13).

The skin is not only a classic source of vitamin D but also a place of synthesis and metabolism of several neuropeptides, including elements of hypothalamic–pituitary–adrenal (HPA) (14), and hypothalamic–pituitary–thyroid (15) axes. The only proopiomelanocortin (POMC) peptides with significant melanogenic activity play a fundamental role in the regulation of melanogenesis, including adrenocorticotrophic hormone (ACTH), α-melanocyte-stimulating hormone (MSH), and β-MSH (16). Several studies have suggested that vitiligo is frequently associated with many other autoimmune diseases, such as thyroid disease (17).

The aim of the current study is to investigate possible relationship between neuroendocrine disorders and etio-pathology of vitiligo by evaluating plasma and serum levels of norepinephrine (NE), epinephrine (Epi), dopamine (DA), homo-vanillic acid (HVA), serotonin (5-HT), 5-hydroxyindoleacetic acid (5-HIAA), melatonin, free triiodothyronine (fT_3_), free thyroxine (fT_4_), thyroid stimulation hormone (TSH), estradiol, testosterone, prolactin, cortisol, and ACTH in blood samples of active and stable vitiligo subjects in a comparative study with healthy subjects.

## Materials and Methods

### Patients and Controls

Informed consent was obtained from 120 volunteers of both sexes. 20 out of 120 patients were excluded from the current study according to exclusion criteria and study limitation as mentioned later in material and methods parts. The included 100 volunteers were divided into two main groups; P, patient group (*n* = 60) and C, healthy control (*n* = 40) groups. Patients and controls were visited by dermatologist (at specialized vitiligo outpatient service between January 2017 and August 2017, Dermatology, Andrology & STDs department, University Hospital, Faculty of medicine, Al-Azhar University, Assuit branch), to confirm the existence or absence of vitiligo.

Sixty acrofacial VP of both sexes were subdivided into two subgroups as active vitiligo patient (AVP), active (*n* = 35; 15 male and 20 female with duration average 2–4 months) and stable vitiligo patient (SVP), stable (*n* = 25); 10 male and 15 female with stabilized disease from at least 2 years. Male subjects (*n* = 25), ranged in age (mean ± SEM = 34.76 ± 2.05 years; minimum = 21 years; maximum = 46 years, median = 35 years) and females (*n* = 35) ranged in age (mean ± SEM = 32.49 ± 1.48 years; minimum = 22 years; maximum = 44 years, median = 33 years). Their BMI was recorded (25 male; mean ± SEM = 23.24 ± 0.54; minimum = 20; maximum = 30, median = 23, and 35 female; mean ± SEM = 24.17 ± 0.30; minimum = 20; maximum = 29, median = 24).

The activity, or progression, of vitiligo was defined as development of new lesions or extension of old lesions during the 3 months before examination by a questionnaire (Do you think that vitiliginous patches are spreading and newly formed now?) on the history of the disease taken during each patient’s first visit to department of Dermatology, Andrology & STDs. The duration of vitiligo and family history of vitiligo were noted and extent of the vitiligo lesions was scored.

Forty healthy subjects includes 15 male ranged in age (mean ± SEM = 30.73 ± 2.48 years; minimum = 18 years; maximum = 42 years, median = 33 years) and 25 female ranged in age (mean ± SEM = 32.04 ± 2.01 years; minimum = 20 years; maximum = 44 years, median = 33 years) without any systemic or dermatological disease, were used as controls. Their BMI was recorded (15 male; mean ± SEM = 21.13 ± 0.68; minimum = 17; maximum = 27, median = 21, and 25 female; mean ± SEM = 22.68 ± 0.67; minimum = 16; maximum = 29, median = 23). Sex and age were matched between the VPs and healthy control groups. Almost all the controls were companions of patients to the centers.

### Exclusion Criteria and Study Limitation

Patients were excluded if had any chronic hepatic, sleep, brain, and/or kidney disorders, seizure. The post-menopausal, pregnant as well as breast feeding women, which have already changes ordinary serum hormone concentrations also were excluded. In addition, patients using oral contraceptives, DA receptor blockers, atypical antipsychotics, metoclopramide, methyldopa, and addicts were excluded. 20 out of 120 patients were excluded from the current study according to this criteria and limitation.

All females in patients and controls were in the luteal phase of their menstrual period at blood sampling. Moreover, all the patients were free of medications for at least a week before participating in the study. Both patients and controls were avoided food and beverages known to affect catecholamine metabolism for at least 24 h both before and throughout the sample collection period. Controls neither had exclusion criteria nor had vitiligo diagnosis at the time of study.

### Preparation of Blood Samples

Fasting 12 ml of blood were taken from the cubital median vein of the patients and controls and divided into two aliquots (6 mL for each). Circadian variations were avoided by always drawing samples between 8:00 a.m. and 9:00 a.m.

#### Serum

The first aliquot was immediately centrifuged 2,000 rpm for 10 min at 4°C. Supernatant serum was divided into eight micro tubes and was separately stored in −80°C before measurement of serum fT_3_, fT_4_, TSH, ACTH, cortisol, estrogen, testosterone, and prolactin levels by ELISA.

#### Plasma

The second aliquot placed into tubes was washed with heparin. The blood samples were centrifuged at 1,000 rpm for 10 min at 4°C. The upper plasma phases were carefully pipette and transferred into seven polypropylene micro tubes and stored at −40°C until assay. Plasma levels of NE, Epi, DA, HVA, serotonin, and 5-HIAA were evaluated by high-pressure liquid chromatography and electrochemical detection (HPLC-ECD). The plasma samples not subjected to more freeze–thaw cycles once they have been prepared readily for the HPLC assay. Melatonin was estimated by ELISA kit.

### Methods

#### Determination of Melatonin, fT_3_, fT_4_, TSH, Estradiol, Testosterone, Prolactin, Cortisol, and ACTH

Serum prolactin was measured by prolactin Human ELISA kit (Cat. No. ab108655, Abcam, UK), melatonin was measured by melatonin ELISA kit (Cat. No. ab213978, Abcam, UK), TSH was measured by TSH Human ELISA kit (Cat. No. ab108660, Abcam, UK), fT_3_ was measured by fT_3_ Human ELISA kit (Cat. No. ab108663, Abcam, UK), fT_4_ was measured by fT_4_ Human ELISA kit (Cat. No. ab108686, Abcam, UK), Estradiol was measured by Estradiol E2 Human ELISA kit (Cat. No. ab108640, Abcam, UK), testosterone was measured by testosterone Human ELISA kit (Cat. No. ab174569, Abcam, UK), Cortisol was measured by Cortisol Human ELISA kit (Cat. No. ab108665, Abcam, UK), and ACTH was measured by Human ACTH ELISA kit (Cat. No. MBS2511227, MyBioSource. Inc., San Diego, CA, USA).

#### Determination of Catecholamines, Serotonin, and Their Metabolites

Plasma levels of NE, Epi, DA, HVA, serotonin, and 5-HIAA were measured by HPLC-ECD (18), after de-proteinization of plasma samples with perchloric acid. Chromatographic separation was carried out on Column—HR-80 (RP—C18), 4.6 mm × 80 mm; 3 mm; 120A (ESA, Inc.). The mobile phase consisted of 100 mM LiH2PO4, 1.5 mM 1-octanesulfonic acid (OSA, ion-pair reagent, SigmaUltra, Sigma-Aldrich), 10% (v/v) methanol (HPLC grade, J. T. Baker, Phillipsburg, NJ, USA) and was delivered at a flow rate of 1.0 mL/min. The electrochemical detector (ECD), Coulochem II-ESA Model 5200A equipped with conditioning (ESA Model, 5021) and analytical cell (ESA Model, 5011), was used at applied potential (millivolts); conditioning cell = +10; analytical cell, E1 = +50; E2 = +340. Recoveries of the assay were between the range of 96 and 105% and the precision (six repeats) was between the ranges of 2.0 and 3.6%.

Some of parameter units were converted from the units provided in manufacturer’s product datasheet to these units, such as fT_3_.

### Statistical Analysis

All results were expressed as mean ± SEM. Statistical analysis for all studied parameters were performed using a one-way analysis of variance followed by Bonferroni posttests multiple comparison tests [vs. Controls and vs. SVP between columns (male vs. male, female vs. female, and/or total vs. total) and within each group (male vs. female)]. All analyses, statistical calculations, and the graph were performed using computer program GraphPad Prism Software version 5.0 (GraphPad Software, San Diego, CA, USA). The values were considered as statistically significant when *p* < 0.05.

## Results

**Figure 1:**
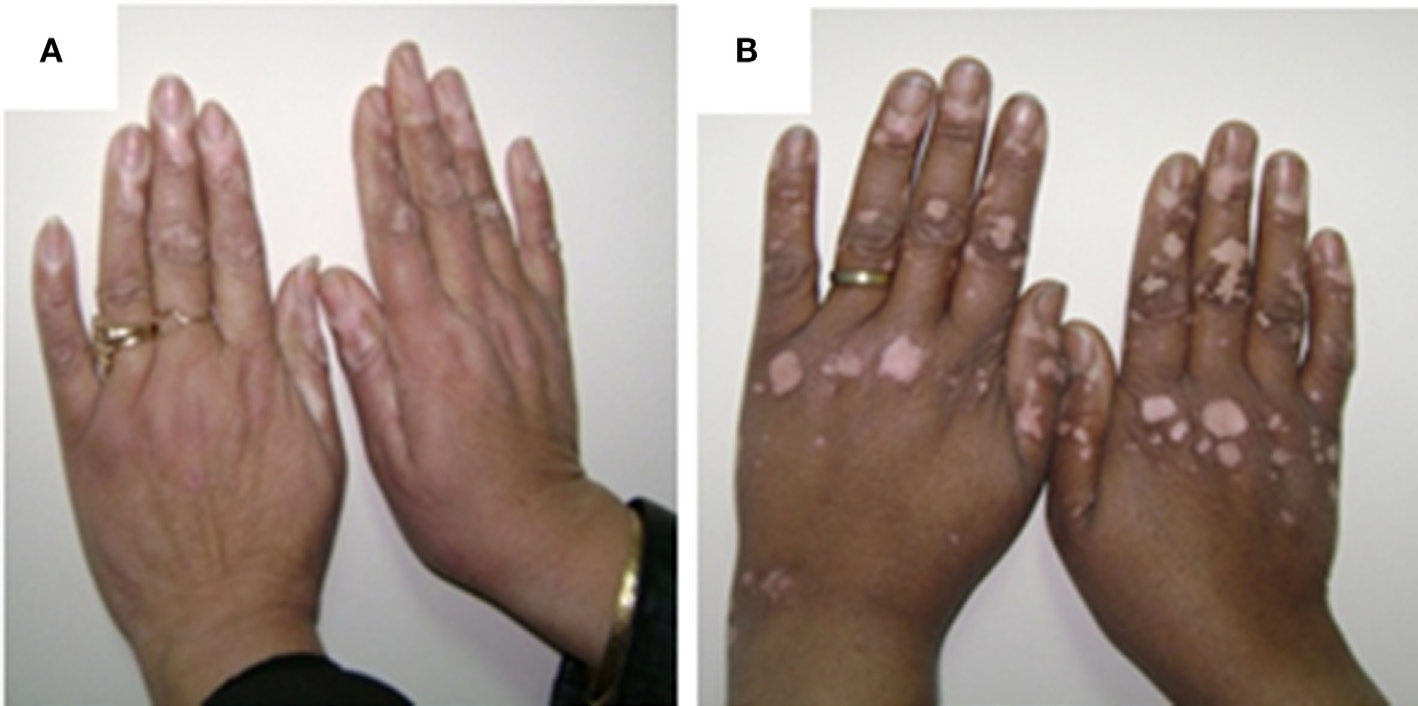
The pictures of female patient, 33 years old showing acral active vitiliginous lesions in both hands **(A)** and female patient, 40 years old showing acral stable vitiliginous lesions in both hands **(B)**. The written informed consent obtained from the participants for publication of the images in this figure.

**Figure 2:**
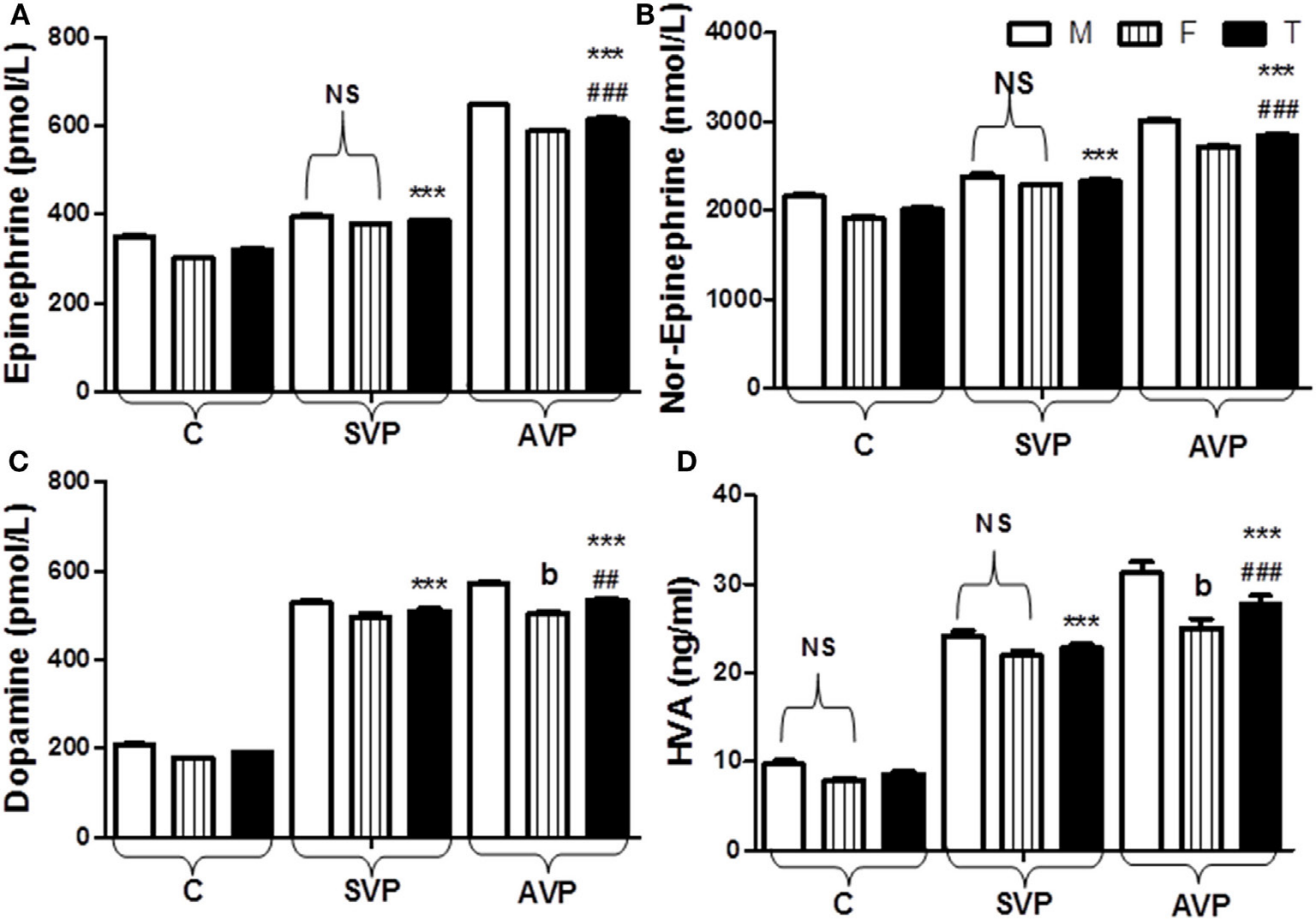
Plasma levels of epinephrine **(A)**, norepinephrine **(B)**, dopamine **(C)**, and HVA **(D)** in vitiligo patients compared with control subjects. Values are expressed as mean ± SEM. ***p < 0.001 significant vs. Control. ^##^*p* < 0.01, ^###^*p* < 0.001 significant vs. SVP, b; non-significant vs. SVP (between groups Total vs. Total; Male vs. Male; Female vs. Female). NS is a non-significant within group (Male vs. Female). Abbreviations: C, control; SVP, stable vitiligo patient; AVP, active vitiligo patient; M, male; F, female; T, total; HVA, homo-vanillic acid.

The current study was limited to active and stable acrofacial VP (Figure 1). Individual data are listed in supplemental table. The data of the current study showed the significant increase in plasma levels of both Epi and NE either in stable or active vitiligo groups than the control group and in active vitiligo group than stable vitiligo group (*p* < 0.001), their levels in males were higher than females within all groups except within a stable vitiligo group (Table 1; Figure 2). The plasma levels of both DA and HVA were significantly increased either in stable or active vitiligo groups than the control group (*p* < 0.001) and in active than stable vitiligo group (*p* < 0.01 and *p* < 0.001, respectively), but there was no significant difference between their levels in females of active group when compared with females of stable vitiligo group (b). Their levels in males were higher than females within all groups, but no effect of the sex on HVA levels within in both stable vitiligo group and control groups (Table 1; Figure 2).

**Table 1:** Plasma levels of epinephrine, NE, DA, HVA, serotonin, 5-HIAA, and melatonin in vitiligo patients compared with control subjects.

Variables	Control group (*n* = 40)			Vitiligo group (*n* = 60)								
				SVP			AVP		
	Male (*n* = 15)	Female (*n* = 25)	Total (*n* = 40)	Male (*n* = 10)	Female (*n* = 15)	Total (*n* = 25)	Male (*n* = 15)	Female (*n* = 20)	Total *(n* = 35)
Epinephrine (pmol/L)	350 ± 3.5	301.6 ± 2.08	319.7 ± 4.17	396.2 ± 3.77	379.2 ± 1.32 (NS)	386 ± 2.37***	647.7 ± 2.89	588 ± 4.62	613.5 ± 5.83^***, ###^
Norepinephrine (nmol/L)	2157 ± 23.22	1920 ± 21.59	2009 ± 24.26	2385 ± 31.53	2288 ± 12.16 (NS)	2327 ± 17.20***	3007 ± 23.60	2718 ± 16.01	2842 ± 27.94***, ^###^
Dopamine (pmol/L)	208.6 ± 2.17	179.6 ± 1.70	190.5 ± 2.60	528.1 ± 4.55	496.1 ± 8.34	508.9 ± 6.14***	570.5 ± 3.01	504.3 ± 4.65^a^	532.7 ± 6.33***, ^##^
HVA (ng/mL)	9.73 ± 0.39	7.92 ± 0.27 (NS)	8.60 ± 0.26	24.10 ± 0.60	21.93 ± 0.56 (NS)	22.80 ± 0.46***	31.27 ± 1.21	25.05 ± 1.02^a^	27.71 ± 0.93***, ^###^
Serotonin (ng/mL)	24.93 ± 0.73	34.16 ± 0.75	30.70 ± 0.89	55.10 ± 1.21	64.93 ± 0.94	61.0 ± 1.22***	74.53 ± 1.61	83.65 ± 1.15	79.74 ± 1.21***, ^###^
5-HIAA (ng/mL)	4.33 ± 0.15	6.0 ± 0.26 (NS)	5.37 ± 0.21	8.30 ± 0.51	9.80 ± 0.48 (NS)	9.20 ± 0.83***	10.60 ± 0.51^a^	12.30 ± 0.31 (NS)	11.57 ± 0.31***, ^###^
Melatonin (ng/mL)	1.46 ± 0.08	2.46 ± 0.05 (NS)	2.09 ± 0.08	4.90 ± 0.23	6.86 ± 0.44	6.08 ± 0.34***	7.0 ± 0.32	8.65 ± 0.43	7.94 ± 0.31***, ^###^

Values expressed as mean ± SEM; ***p < 0.001 and means a significant difference between SVP or AVP groups and control group while ^##^*p* < 0.01 and ^###^*p* < 0.001 and means a significant difference between AVP and SVP group (Total vs. Total; between groups).

*^a^Means a non-significant difference between AVP and SVP (Total vs. Total; Male vs. Male; Female vs. Female, between groups)*.

*NS means a non-significant difference within groups (Male vs. Female)*.

*SVP, stable vitiligo patients; AVP, active vitiligo patient; HVA, homo-vanillic acid; 5-HIAA, 5-hydroxyindolacetic acid; NE, norepinephrine; DA, dopamine*.

**Figure 3:**
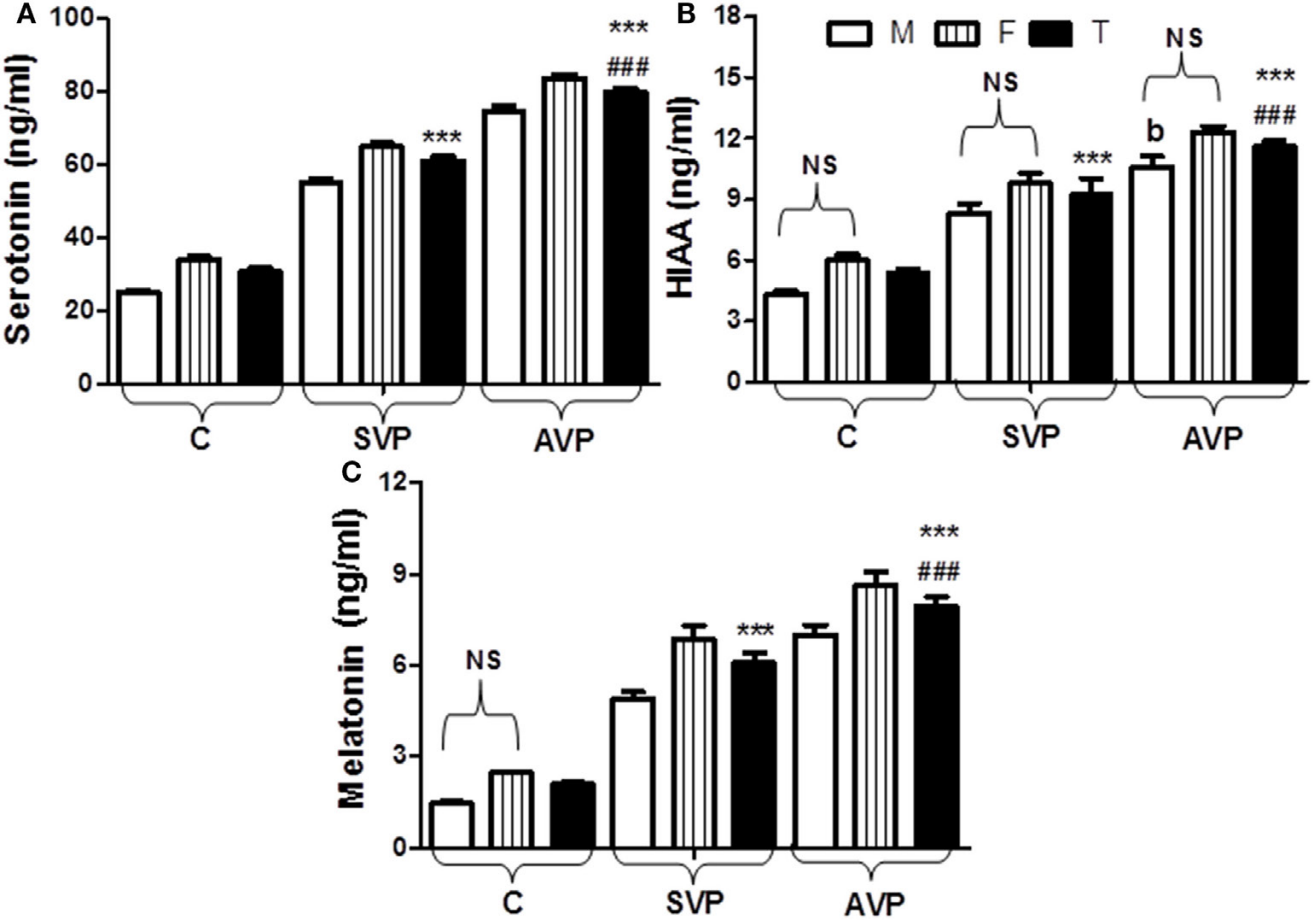
Plasma levels of serotonin **(A)**, 5-HIAA **(B)**, and melatonin **(C)** in vitiligo patients compared with control subjects. Values expressed as mean ± SEM. ***p < 0.001 significant vs. Control. ^###^*p* < 0.001 significant vs. SVP, b; non-significant vs. SVP (between groups Total vs. Total; Male vs. Male; Female vs. Female). NS; is a non-significant within group (Male vs. Female). Abbreviations: C, control; SVP, stable vitiligo patient; AVP, active vitiligo patient; M, male; F, female; T, total; 5-HIAA, 5-hyroxyindoleacetic acid.

The plasma levels of serotonin, 5-HIAA, and melatonin were significantly increased either in stable vitiligo group or active vitiligo group than the control group (*p* < 0.001) and in active vitiligo group than stable vitiligo group (*p* < 0.001). The levels of both serotonin and melatonin in females were significantly higher than males within all groups, while no effect of the sex on 5-HIAA levels within all studied groups. There was no significant difference between 5-HIAA levels in males of active vitiligo group when compared with males of stable vitiligo group (b) (Table 1; Figure 3).

**Figure 4:**
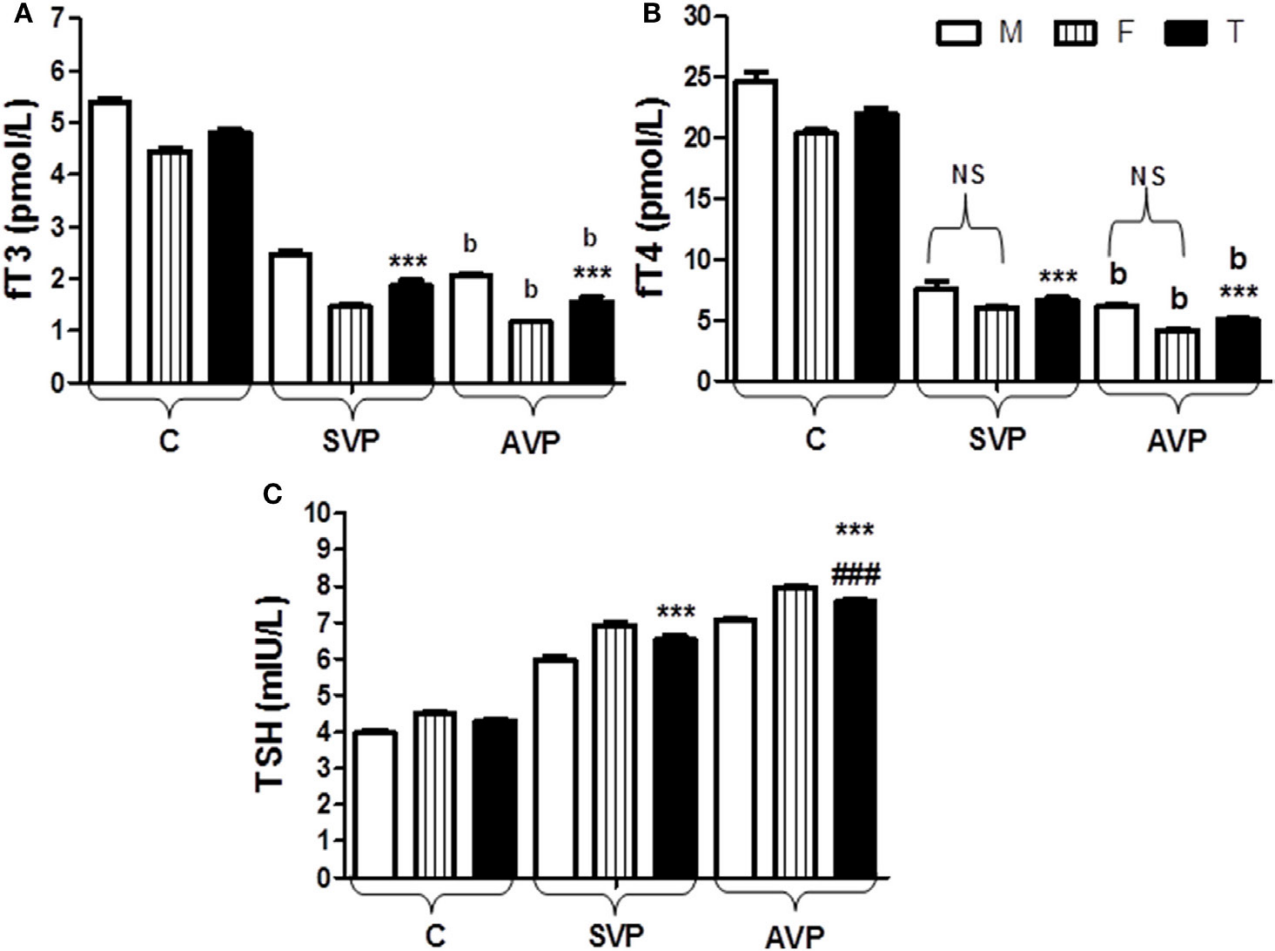
Serum levels of fT_3_ **(A)**, fT_4_ **(B)**, and TSH **(C)** in vitiligo patients compared with control subjects. Values expressed as mean ± SEM. ***p < 0.001 significant vs. Control. ^###^*p* < 0.001 significant vs. SVP, b; non-significant vs. SVP (between groups Total vs. Total; Male vs. Male; Female vs. Female). NS is a non-significant within group (Male vs. Female). Abbreviations: C, control; SVP, stable vitiligo patient; AVP, active vitiligo patient; M, male; F, female; T, total; fT_3_, free triiodothyronine; fT_4_, free thyroxine; TSH, thyroid-stimulating hormone.

The serum levels of fT_3_ and fT_4_ were significantly decreased either in stable vitiligo group or active vitiligo group than the control group (*p* < 0.001) while no significant difference (b) between active vitiligo group and stable vitiligo group groups. The levels fT_3_ in males were significantly higher than females within all groups, while no effect of the sex on fT_4_ levels within all studied groups. The serum levels thyroid-stimulating hormone (TSH) was significantly increased either in stable vitiligo group or active vitiligo group than the control group and in active vitiligo group than stable vitiligo group (*p* < 0.001). The levels TSH in females were significantly higher than males within all (Table 2; Figure 4).

**Table 2:** Serum levels of fT_3_, fT_4_, TSH, estradiol, testosterone, prolactin, cortisol, and ACTH in vitiligo patients compared with control subjects.

Variables	Control group (*n* = 40)			Vitiligo group (*n* = 60)					
				SVP			AVP		
	Male (*n* = 15)	Female (*n* = 25)	Total (*n* = 40)	Male (*n* = 10)	Female (*n* = 15)	Total (*n* = 25)	Male (*n* = 15)	Female (*n* = 20)	Total (*n* = 35)
fT_3_ (pmol/L)	5.38 ± 0.08	4.45 ± 0.06	4.80 ± 0.08	2.45 ± 0.09	1.46 ± 0.06	1.86 ± 0.11***	2.07 ± 0.04^b^	1.17 ± 0.02^b^	1.56 ± 0.08^b, ***^
fT_4_ (pmol/L)	24.67 ± 0.76	20.36 ± 0.36	21.98 ± 0.48	7.55 ± 0.62	6.03 ± 0.06 (NS)	6.64 ± 0.28***	6.20 ± 0.07^b^	4.16 ± 0.14^b^ (NS)	5.03 ± 0.19^b^ ***
TSH (mIU/L)	3.98 ± 0.07	4.5 ± 0.06	4.30 ± 0.06	5.97 ± 0.11	6.93 ± 0.10	6.54 ± 0.12***	7.06 ± 0.07	7.96 ± 0.06	7.57 ± 0.09^***, ###^
Estradiol (pmol/L)	106.7 ± 1.94	1250 ± 4.54	821.5 ± 88.71	124.8 ± 1.10^a^	1302 ± 8.11^a^	831.1 ± 117.8^a^	165.1 ± 1.06^a,b^	2103 ± 32.83	1272 ± 165.5^b,^ *
Testosterone (nmol/L)	25.2 ± 0.79	1.54 ± 0.04	10.42 ± 1.85	9.90 ± 0.52	2.06 ± 0.20^a^	5.20 ± 0.81*	2.49 ± 0.03	0.98 ± 0.04^a,b^ (NS)	1.62 ± 0.13^b***^
Prolactin (μg/L)	7.95 ± 0.32	20.76 ± 0.59	15.96 ± 1.06	22.4 ± 0.65	34.53 ± 0.68 (NS)	29.68 ± 1.30***	42.67 ± 0.74	85.15 ± 1.23	66.94 ± 3.68^***, ###^
Cortisol (nmol/L)	250.4 ± 0.88	454.2 ± 2.29	377.8 ± 15.87	311 ± 4.33^a^	473 ± 3.89^a^	408.2 ± 16.45^a^	710.3 ± 5.82	865.8 ± 4.69	799.1 ± 13.67^***, ###^
ACTH (pmol/L)	7.92 ± 0.21	5.0 ± 0.22	6.10 ± 0.27	4.65 ± 0.17	2.42 ± 0.06	3.31 ± 0.23***	2.82 ± 0.03	1.53 ± 0.05^b^	2.08 ± 0.11^***, ###^

*Values expressed as mean ± SEM*.

*p < 0.05, ***p < 0.001 and means a significant difference between SVP or AVP groups and control group while ^###^*p* < 0.001, means a significant difference between AVP and SVP groups (Total vs. Total; between groups).

*^a^Means a non-significant difference between SVP or AVP group and control group*.

*^b^Means a non-significant difference and AVP and SVP (Total vs. Total; Male vs. Male; Female vs. Female, between groups)*.

*NS means a non-significant difference within groups (Male vs. Female)*.

*SVP, stable vitiligo patient; AVP, active vitiligo patient; fT_3_, free triiodothyronine; fT_4_, free thyroxine; TSH, thyroid-stimulating hormone; ACTH, adrenocorticotrophic hormone*.

**Figure 5:**
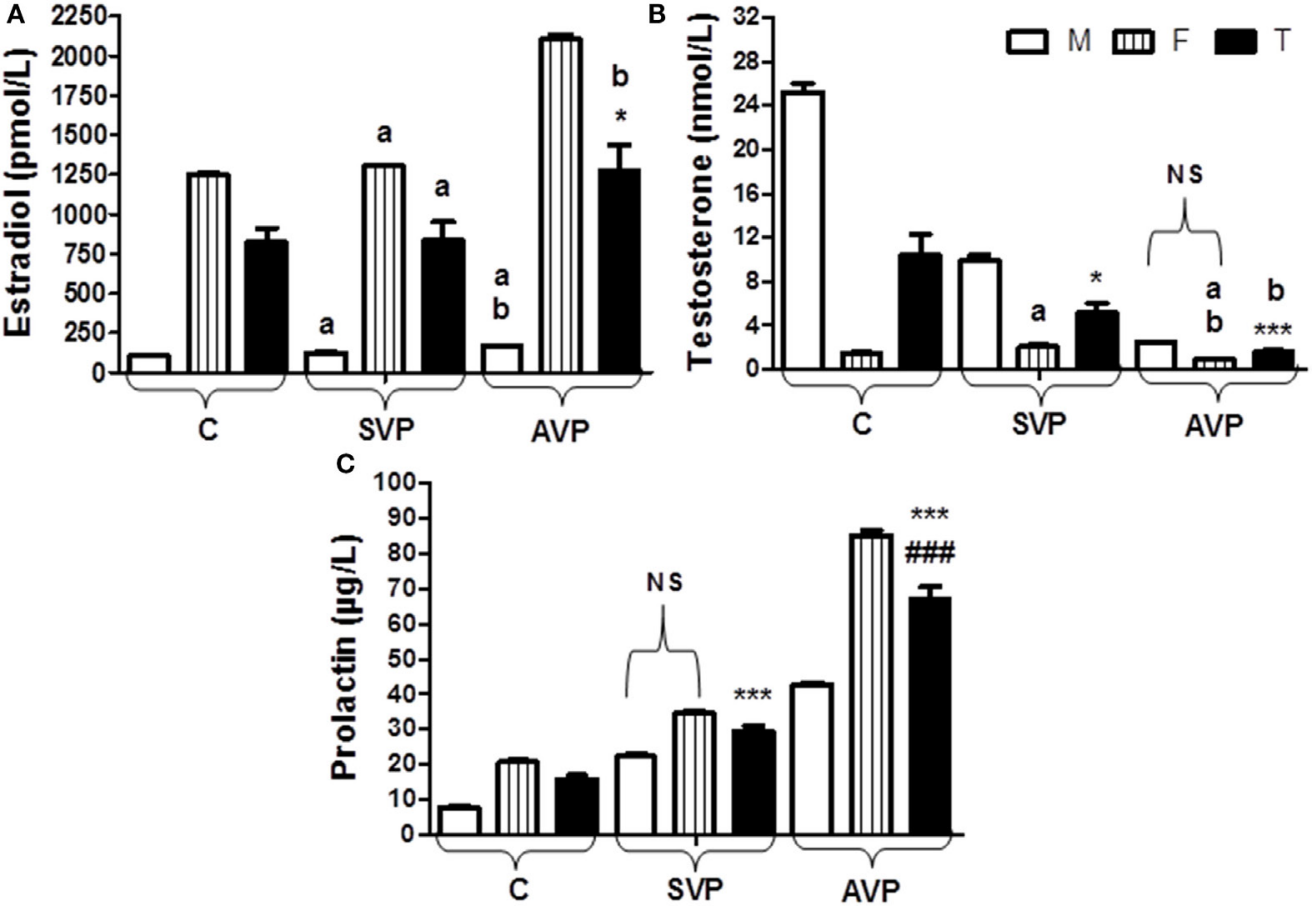
Serum levels of estradiol **(A)**, testosterone **(B)**, and prolactin **(C)** in vitiligo patients compared with control subjects. Values expressed as mean ± SEM. *p < 0.05, ***p < 0.001 significant vs. Control. ^###^*p* < 0.001 significant vs. SVP, a; non-significant vs. control, b; non-significant vs. SVP (between groups Total vs. Total; Male vs. Male; Female vs. Female). NS is a non-significant within group (Male vs. Female). Abbreviations: SVP, stable vitiligo patient; AVP, active vitiligo patient; M, male; F, female; T, total.

There was no significant difference in estradiol levels when either stable vitiligo group or active vitiligo group compared with control group (a), or when active vitiligo group compared with stable vitiligo group, except a significant increase in estradiol levels was observed in females within active vitiligo group than females in either stable vitiligo group or control groups. There was a significant decrease in testosterone levels in serum of males in either active vitiligo group or stable vitiligo group when compared to controls, and in active vitiligo group than stable vitiligo group; therefore, there was no significant difference in testosterone level between males and females in the active vitiligo group (Table 2; Figure 5).

The serum prolactin level was significantly increased in either stable vitiligo group or active vitiligo group when compared with control and in active vitiligo group than stable vitiligo group (*p* < 0.001) with non-significant difference in prolactin levels between males and females within a stable vitiligo group (Table 2; Figure 5).

**Figure 6:**
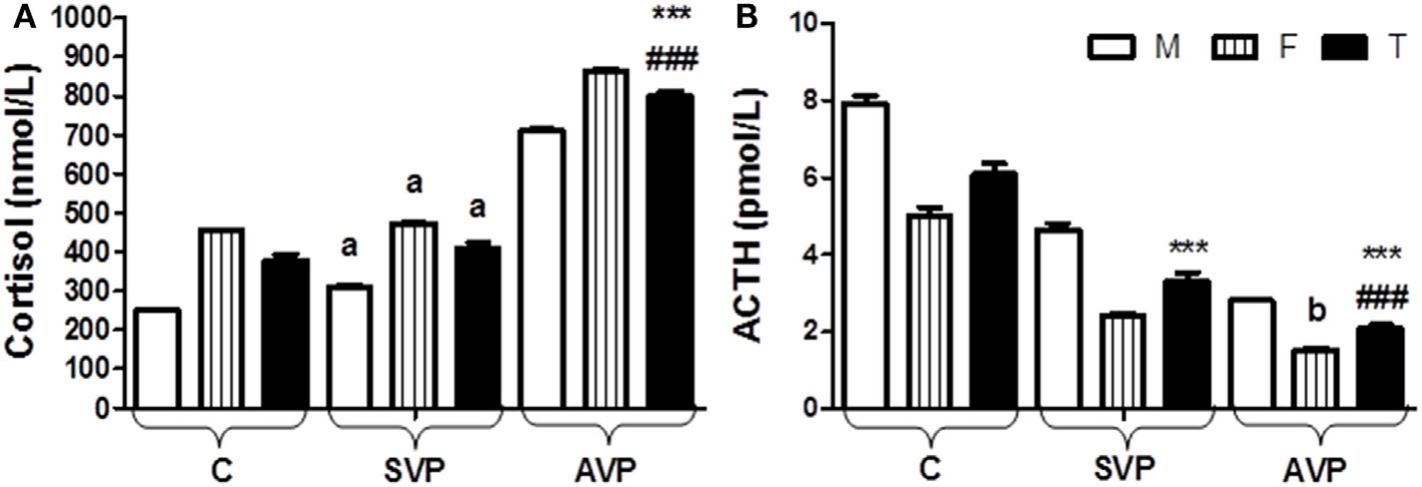
Serum levels of cortisol **(A)** and ACTH **(B)** in vitiligo patients compared with control subjects. Values expressed as mean ± SEM. ***p < 0.001 significant vs. Control. ^###^*p* < 0.001 significant vs. SVP, a; non-significant vs. control, b; non-significant vs. SVP (between groups Total vs. Total; Male vs. Male; Female vs. Female). Abbreviations: SVP, stable vitiligo patient; AVP, active vitiligo patient; M, male; F, female; T, total; ACTH, adrenocorticotrophic hormone.

There was no any significant difference in serum level of cortisol between stable vitiligo group and control group (a), while there was a significant increase in serum level of cortisol in active vitiligo group than either stable vitiligo group or control group (*p* < 0.001). Within all studied groups, the cortisol levels were higher in females than males. There was a significant decrease in serum level of ACTH in either active vitiligo group or stable vitiligo group than control and in active vitiligo group than stable vitiligo group (*p* < 0.001). Within all studied groups, the ACTH levels were higher in males than females (Table 2; Figure 6).

## Discussion

The etiology of vitiligo is still complexed and unclear. Several theories were proposed to explain the loss of melanocyte function (19). The current study focused on a neural and hormonal hypothesis. As consequences of neural and endocrinal disturbances, we will discuss here other interrelated causes including; an autoimmune mechanism, an altered tetrahydrobiopterin (BH_4_) homeostasis (5), psychological stressors (6), and defective-free radical defense on melanin production (9).

Neural factors as well as emotional and/or stressful events seem to play a pivotal role in vitiligo onset or exacerbation. Schallreuter (20) revealed that human keratinocytes are fully able to synthesize and degrade catecholamines. Tyrosine was converted into melanin and catecholamine type neurotransmitters (neural signaling molecules DA, NE, Epi, etc., that control both central and peripheral nervous systems by a tyrosine hydroxylase enzyme) (21).

The results of the current study showed the significant increase in plasma levels of both Epi and NE in vitiligo groups than controls but, in active than stable vitiligo, their levels in males were higher than females within all groups except within stable vitiligo (Table 1; Figure 2). Many studies of VP have confirmed this neural hypothesis. The depigmented areas tend to sweat less, and to have different temperature regulation, electrical resistance, and other related neural dysfunctions in the skin (20). So, this increased level of Epi and NE in VP, possibly due to a stress-induced secretion and their levels may play a role in appearance of depigmented patches as reported in many studies irrespective of the type of vitiligo (22).

In context, the relationship between increased catecholamine levels and depigmentation was illustrated by a biochemical hypothesis [an abnormal function of the metabolic system of biopterins related to high levels of the (6R)-l-erythro 5,6,7,8-tetrahydrobiopterin (6BH_4_) and its isomer 7BH4 in vitiligo epidermis related to increased catecholamine biosynthesis on expense of melanin and formation of micromolar levels of H_2_O_2_, which is toxic for melanocytes (23)] and by autoimmune hypothesis (where a damaged melanocyte triggers an autoimmune reaction bringing on its own destruction) (4). Moreover, biopterins act as inhibitors of the enzymes involved in melanogenesis (namely, phenylalanine hydroxylase and tyrosinase) (24).

In context, stress products such as reactive oxygen species (ROS) can be produced by exogenous and endogenous stimuli such as catecholamine. In addition, abnormally increased catecholamine can produce vasoconstriction leading to epidermal–dermal hypoxia, and possibly oxidized by different oxidative systems with formation of quinones, semiquinone radicals, and oxyradicals. However, local and systemic high levels of H_2_O_2_ produced by catecholamine are able to alter calcium homeostasis, so perturbing the uptake of l-phenylalanine, the amino acid precursor of tyrosine in melanocytes. It is reasonable to suggest that the increased levels of these oxidative radicals from oxidation of monoamine and their metabolites might contribute to melanocyte damage in the early phase of vitiligo (24, 25).

The plasma levels of both DA and HVA were significantly increased either in vitiligo groups than the control group but in active than stable group. Their levels in male were higher than female within all groups, but no effect of the sex on HVA levels within in both SVP and control groups (Table 1; Figure 2). Irrespective of the type of vitiligo, the current results in agreement with many studies (22), but in patients at recent onset of the disease than chronic sufferers.

DA is a well-known neurotoxin inducing oxidative stress and is thought to be the most potent toxic monoamine (compared with NE, Epi, and 5-HT) in inducing apoptosis of neuronal cells as well as in human melanocytes (26). This demonstrates the relationship between increased DA level and vitiligo.

In contrast to the results of the current study and the abovementioned studies, Orecchia et al. (27) failed to find significant increases in the levels of plasma catecholamines and their metabolites in patients with generalized and acrofacial vitiligo. However, these patients were not subdivided according to the recent onset (<1 year) or progression of disease.

Skin cells have fully functional serotonin and melatoninergic systems and express steroidogenic activity. Melatonin modulates melanogenesis (11) and exhibits a number of properties that involved in stress–response system of the skin (28). There is a complex relationship between skin pigmentary function and 5-HT.

The plasma levels of serotonin, 5-HIAA, and melatonin in the current study were significantly increased either in vitiligo groups than the control group and in active than stable group. The levels of both serotonin and melatonin in females were significantly higher than males within all groups while no effect of the sex on 5-HIAA levels within all studied groups (Table 1; Figure 3).

The current results in the same line with many investigators suggested that melanin synthesis was disrupted by anomalies of the melatonin receptor. The activated receptors of—or the increased output of—melatonin at peripheral nerve endings in the skin lightens the color of pigment cells and decreases new melanin formation causing vitiligo (29). So, melatonin can inhibit melanin synthesis without affecting the first steps of melanogenesis (30). Abnormal activation of the melatonin receptor can occur because of an increased release of catecholamines and other neurotransmitters (31).

In the same line with the current study, the higher 5-HIAA levels, also noted by Chakraborty et al. (32). in the urine of vitiliginous patients, can be explained by considering that (a) this metabolite of 5-HT is a product of monoamine oxidase (MAO)-A activity; (b) another MAO-A compound (HVA) showed significantly higher levels in VP; and (c) a significant relationship between 5-HIAA and HVA was found. Thus, these data would further support the hypothesis of increased MAO-A activity in vitiligo and in agreement with current results of serotonin, 5-HIAA, and HVA. However, 5-HT dose dependently inhibits melanin production and tyrosinase activity in human SK-MEL-188 melanoma cells (33). In contrast to the current study, the content of 5-HT in blood was decreased in patients with vitiligo as compared with healthy persons (34).

In literature, circulating antibodies to melanocytes were detected in VP (35). Vitiligo is associated with autoimmune disorders; this indirectly supports the idea of an autoimmune pathogenesis of the disease (36). Prolactin seemed to have more functions in skin biology and hair follicle growth as well (37). The serum prolactin level was significantly increased in either SVP or AVP when compared with control and in AVP than an SVP group with non-significant difference in prolactin levels between males and females within an SVP group (Table 2; Figure 5). The result of the current study supports the link between endocrine hypothesis and autoimmune hypothesis in vitiligo etio-pathology. Prolactin is a growth factor for lymphocytes with the potential to stimulate immune responses against melanocytes (38).

The disturbance of melanocyte homeostasis due to eczema, vitiligo, and during pregnancy is should be considered for illustration of real pathogenesis of skin depigmentation (39). In the current study, there was no significant difference in estradiol levels when either SVP or AVP compared with control group (a), or when AVP compared with SVP, except significant increase in estradiol levels was observed in females within AVP than females in either SVP or control groups (Table 2; Figure 5). Hence, presence of oxidative stress particularly H_2_O_2_ increase in the skin and even in blood cells of VP as stated by Rokos et al. (40), is possibly due to increased levels of estrogens (as observed in the current study) and this possibly involved in the depigmentation process of vitiligo as reported by Anderson et al. (41). In addition, an important enzymes, such as BH_2_ reductase (EC 1.6.99.7), which was involved in the recycling of the important cofactor (6R)-l-erythro-5,6,7,8-BH_4_ and was deactivated through oxidation of important residues in its structures by H_2_O_2_ (23, 24).

However, melisma (grayish brown facial pigmentation) may often appear in non-pregnant women using oral contraceptive pills which contain only steroid hormone analogs (42), suggesting that skin pigmentation in humans could be controlled by a factor other than α-MSH such as estrogen. So, estrogen acts as ligands and affects melanin pigment production through its receptors on melanocytes (43).

Different from the current results, females at child-bearing age due to high estrogen concentration might be less prone to vitiligo as reported by Kang and Ortonne (44).

The lack of fertility and vitiligo were linked to each other. In male hypogonadism, testosterone hormone production is inadequate, which leads to infertility (45). There was significant decrease in testosterone levels in serum of males in either AVP or SVP when compared to controls, and in AVP than SVP, therefore there was no significant difference in testosterone level between males and females with AVP group (Table 2; Figure 5). Here, a decrease in testosterone levels in VP, possibly due to emotional distress and low self-esteem (46) that affects sexual life of the patients (47).

The skin is not only a classical source of vitamin D but also a place of synthesis and metabolism of several neuropeptides, including elements of hypothalamic–pituitary–thyroid (15) axes. Several studies have suggested that vitiligo is often associated with many other autoimmune diseases, including thyroid disease. Vitiligo commonly coexists with such diseases in a maximum of 20% of cases (48).

The serum levels of fT_3_ and fT_4_ were significantly decreased either in VP than the control group in spite of type of vitiligo. The levels fT_3_ in males were significantly higher than females within all groups while no effect of the sex on fT_4_ levels within all studied groups. The serum levels TSH was significantly increased either in VP than the control group and in AVP than SVP. The levels TSH in females were significantly higher than males within all groups (Table 2; Figure 4).

This decrease in thyroid hormone levels in blood of VP, possibly due to autoimmune hypothyroidism due to a strict interplay between oxidative stress and immune system, affect skin too, as observed in the form of conditions such as dermatitis, urticaria, and vitiligo as reported in many studies (49). These studies show prevalence of thyroglobulin and thyroid peroxidase b antibodies in VP than non-vitiligo controls, but in females than males (50).

In addition, tyrosine is an essential for biosynthesis of thyroid hormone, is its deficiency leading to thyroid malfunction? From abovementioned results about increased catecholamines levels in VP’ blood, it was predicted that thyroid hormones levels decreased due to an increase in catecholamine biosynthesis from tyrosine on expense of thyroid hormones. Moreover, an abnormal level of testosterone has a role in hypothyroidism in studied vitiligo groups as reported by Bahrami et al. (51) who stated that testosterone has beneficial effect on thyroid functions in rodent model. In the same line with the results of the current study, Biswas et al. (52) conclude that vitiligo usually precedes the onset of thyroid dysfunction. Kasumagic-Halilovic et al. (17) reported significantly higher frequency of autoimmune thyroiditis in girls and in the patients with long duration of generalized vitiligo.

On the other hand and with disagreement with the current results, the vitiligo correlated with the thyroid disturbance such as Hashimoto’s disease and Graves’ disease is very strong (53).

The skin is not only a classic source of vitamin D but also a place of synthesis and metabolism of several neuropeptides, including elements of HPA (14) axes.

Stress causes the release of corticotrophin-releasing factor (CRF) from brain hypothalamus and skin. Binding of CRF to the receptors on pituitary gland stimulates production of POMC and its further proteolytic processing to adrenocorticotropin (ACTH), α-MSH, lipotropins (LPH), and β-endorphin, all are secreted by anterior pituitary and ACTH is the prominent hormone responding to stress. ACTH stimulates the adrenal cortex to produce cortisol, a glucocorticoid (54). Melanin synthesis is regulated by α-MSH elaborated from POMC (55). So, it can be concluded that melanin synthesis is a process regulated by an array of hormones, and disruption of any of them can interrupt the pigment genesis. It has long been known that MSH and ACTH play a fundamental role in regulation of melanogenesis (56). On the other hand, MSH activates melanin synthesis in melanocytes by interaction of MC1R receptor (57).

The current results showed no any significant difference in serum level of cortisol between SVP and control group (a), while there was a significant increase in serum level of cortisol in AVP than either SVP or control group. Within all studied groups, the cortisol levels were higher in females than males. There was a significant decrease in serum level of ACTH in either AVP or SVP than control and in AVP than SVP group. Within all studied groups, the ACTH levels were higher in males than females (Table 2; Figure 6).

From abovementioned results of the current study, we hypothesized that CRF, POMC, ACTH, and MSH possibly feedback inhibited by higher levels of cortisol in VP, so decrease melanin biosynthesis in the skin. In addition, a predicted decreased level of CRF affects the role nuclear factor kappa B (NF-κB) in human keratinocytes as regulator of immune response (58, 59). Moreover, ROS produced by estrogen and catecholamine are able to oxidize and inhibit the activity of POMC-derived bioactive peptides ACTH and α-MSH leading to skin depigmentation.

Considering a comparison of neuro-endocrinal regulation between active and stable VP groups; a significant increase in plasma levels of Epi, NE, DA, HVA, serotonin, 5-HIAA, melatonin, prolactin, cortisol, and TSH was observed in AVP than SVP group. In addition, a significant increase in estradiol levels (in females) and a significant decrease in testosterone levels (in males) were noted within AVP than SVP. Moreover, there was a significant decrease in serum level of ACTH in AVP than SVP group. All these findings lend credence to the hypothesis that active vitiligo melanocytes are more prone to neuro-endocrinal changes than stable vitiligo. The neural hypothesis is compatible with many studies including Morrone et al. (60) and Cucchi et al. (22), who reported significant increases in the plasma and urinary concentrations of the catecholamine metabolites in active VP. The explanation for the differences in the results between stable and active vitiligo subgroups is possibly attributed to the communication between endocrine, nervous, and immune systems through hormonal neuropeptide route and to their adaptation with stress factors causing vitiligo disease for the maintenance of a homeostatic balance within the body and good health. This explanation supported by conclusion of Taub (61). The current study is possibly the first study to observe the endocrinal differences in all studied hormones between the active and stable vitiligo with notation that there was no significant difference between AVP and SVP groups about levels of fT_3_ and fT_4_.

**Figure 7:**
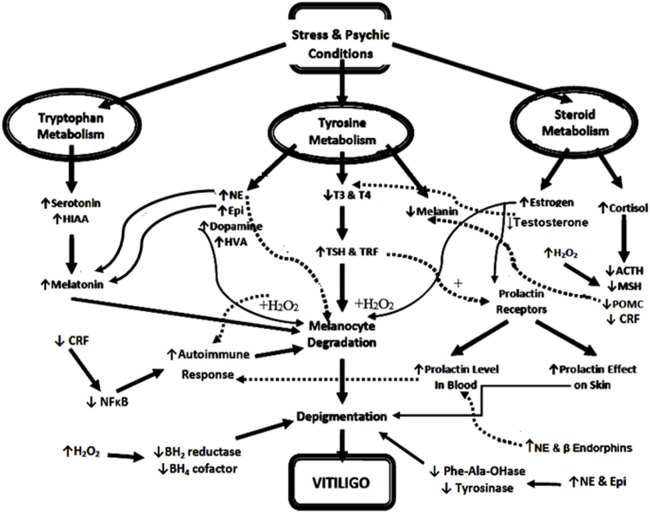
Diagrammatic illustration of possible neuro-endocrinal pathogenesis of vitiligo. Abbreviations: NE, norepinephrine; Epi, epinephrine; T_3_, triiodothyronine; T_4_, thyroxine; TSH, thyroid-stimulating hormone; CRF, corticotrophin-releasing factor; TRF, thyrotrophic-releasing factor; MSH, melanocyte-stimulating hormone; POMC, proopiomelanocortin; NF-κB, nuclear factor kappa B; BH_4_, tetrahydrobiopterin; BH_2_, dihydrobiopterin; ACTH, adrenocorticotrophic hormone; HVA, homo-vanillic acid; HIAA, hydroxyindoleacetic acid; H_2_O_2_, hydrogen peroxide; Phe-Ala-OHase, phenylalanine hydroxylase. ↑ and +, increase or activate, ↓ or −, decrease or inhibits, the solid lines mean above the page plane, whereas the dotted lines mean below page plane to differentiate between the beginning and the end of each arrow.

Based on the previous studies and the data of the current study, we conclude that the pathogenesis of vitiligo is multifactorial owing to psychic stress, autoimmune, endocrinal, neural, oxidative stress, and many biochemical factors. Considering neural and endocrinal hypothesis, the different types of stress stimulates catecholamine synthesis from tyrosine on expense of both melanin and thyroid hormones biosynthesis, leading to skin depigmentation and hypothyroidism. Increased catecholamines stimulate melatonin biosynthesis or activate its receptors on skin resulting in a decrease in melanin biosynthesis. Moreover, increased levels of catecholamines, DA, estrogen, and prolactin (62) result in hyper-production of H_2_O_2_ that leads to either induction of immune response against melanocytes (depigmentation) or inhibition of BH_2_ reductase and BH_4_ cofactor (reduced pigmentation). In addition, stress-induced cortisol hypersecretion is inhibited by feedback regulation of CRF, POMC, ACTH, and MSH related to abnormally low pigmentation of skin and/or the changes of CRF effects on NF-κB immune response, resulting in melanocyte destruction (Figure 7).

The present study possibly introduces a new efficient strategy for attenuation of vitiligo progress and improvement of vitiligo treatment, so we recommend that any dermatologist should take the neurological, psychological, and endocrine disorders into account because the possible causes associated with vitiligo disease are able to control the progress of the disease, turn it quickly from active to stable state and improve vitiligo treatment. Collectively, we can address these neuro-endocrinal disorders associated with vitiligo formation.

## Availability of Data and Materials

All data generated or analyzed during this study included in this published article.

## Ethics Statement

The main aims, the research process, and the value of the findings explained to the participants before obtaining their written informed consents. Investigators kept patients’ and controls’ information as medical secrets. All the test fees and research processes were free of charge for the studied subjects. The study design conducted according to the Declaration of Helsinki principles. The “Medical Research ethics committee” of Dermatology, Andrology & STDs Department (in Faculty of Medicine, Al-Azhar University; Assuit Branch, Egypt) has revised the manuscript protocol and approved the research protocol which following all the ethical regulations stated by the committee. The committee ethical regulations prohibits disclosing names and personal information for any patient without legislative procedure or applying any invasive protocol was not needed for the diagnosis such a tissue biopsy. All blood samples were collected during regular and routine diagnosis procedure for the patients without using any invasive protocols.

## Author Contributions

All authors shared with an equal effort in all study steps, including study design, experimental analysis of the samples, statistical analysis, and manuscript writing and reviewing.

## Conflict of Interest Statement

The authors declare that the research was conducted in the absence of any commercial or financial relationships that could be construed as a potential conflict of interest.

## Abbreviations

AVP, active vitiligo patient; SVP, stable vitiligo patient; T, total; F, female; M, male; fT_3_, free triiodothyronine; fT_4_, free thyroxine; TSH, thyroid-stimulating hormone; ACTH, adrenocorticotrophic hormone; NE, norepinephrine; Epi, epinephrine; DA, dopamine; HVA, homo-vanillic acid; 5-HIAA, 5-hydroxyindoleacetic acid; 5-HT, 5-hyroxytryptamine; MSH, melanocyte-stimulating hormone; H_2_O_2_, hydrogen peroxide; HPLC, high-performance liquid chromatography; BH4, tetrahydrobiopterin; BH_2_, dihydrobiopterin; ROS, reactive oxygen species; MAO, monoamine oxidase; CRF, corticotrophin-releasing factor; POMC, proopiomelanocortin; HPA, hypothalamic–pituitary–adrenal; NF-κB, nuclear factor kappa B.

## References

[1] Kruger C Schallreuter KU. A review of the worldwide prevalence of vitiligo in children/adolescents and adults. Int J Dermatol (2012) 51:1206–12. 10.1111/j.1365-4632.2011.05377.x 22458952

[2] Dell’Anna ML Maresca V Briganti S Camera E Falchi M Picardo M. Mitochondrial impairment in peripheral blood mononuclear cells during the active phase of vitiligo. J Invest Dermatol (2001) 117(4):908–13. 10.1046/j.0022-202x.2001.01459.x 11676831

[3] Kemp EH Waterman EA Weetman AP. Immunological pathomechanisms in vitiligo. Expert Rev Mol Med (2001) 23:1–22. 10.1017/S1462399401003362 14585144

[4] van den Wijngaard R Wankowicz-Kalinska A Pals S Weening J Das P Autoimmune melanocyte destruction in vitiligo. Lab Invest (2001) 81(8):1061–7. 10.1038/labinvest.3780318 11502857

[5] Schallreuter KU Moore J Wood JM Beazley WD Eva M Peters J Epidermal H_2_O_2_ accumulation alters tetrahydrobiopterin (6bh4) recycling in vitiligo: identification of a general mechanism in regulation of all 6BH4-dependent processes? J Invest Dermatol (2001) 116:167–74. 10.1046/j.1523-1747.2001.00220.x 11168813

[6] Silverberg JI Silverberg NB. Vitiligo disease triggers: psychological stressors preceding the onset of disease. Cutis (2015) 95(5):255–62. 26057504

[7] Orecchia GE Neural pathogenesis. In: Hann SK Nordlund JJ, editors. Vitiligo. London: Blackwell Science (2000). 142 p.

[8] Reichert-Faria A Tarlé RG Dellatorre G Mira MT Silva de Castro CC Vitiligo – part 2 – classification, histopathology and treatment. An Bras Dermatol (2014) 89(5):784–90. 10.1590/abd1806-4841.20142717 25184918 PMC4155957

[9] He Y Li S Zhang W Dai W Cui T Wang G Dysregulated autophagy increased melanocyte sensitivity to H_2_O_2_-induced oxidative stress in vitiligo. Sci Rep (2017) 7:42394. 10.1038/srep42394 28186139 PMC5301258

[10] Zmijewski MA Slominski AT. Neuroendocrinology of the skin: an overview and selective analysis. Dermatoendocrinol (2011) 3(1):3–10. 10.4161/derm.3.1.14617 21519402 PMC3051846

[11] Sun X Wang Y Jiang N Du Z Sun H Sun L The potential role of melatonin on mental disorders: insights from physiology and pharmacology. Bipolar Disord (2016) 2(1):105–9. 10.4172/2472-1077.1000105

[12] Radvar SE Tehranchi Z Partovikia M Kazeminejad A Amini SHS Assessment of prolactin levels in vitiligo patients and healthy controls and it’s association with severity of disease. Pigm Disord (2014) 2:155 10.4172/2376-0427.1000155

[13] Schallreuter KU Chiuchiarelli G Cemeli E Elwary SM Gillbro JM Spencer JD Estrogens can contribute to hydrogen peroxide generation and quinone-mediated DNA damage in peripheral blood lymphocytes from patients with vitiligo. J Invest Dermatol (2006) 126:1036–42. 10.1038/sj.jid.5700257 16528352

[14] Jozic I Stojadinovic O Kirsner RSF Tomic-Canic M Skin under the (Spot)-light: crosstalk with the central hypothalamic–pituitary–adrenal (HPA) axis. J Invest Dermatol (2015) 135:1469–71. 10.1038/jid.2015.56 25964265

[15] Safer JD. Thyroid hormone action on skin. Dermatoendocrinol (2011) 3(3):211–5. 10.4161/derm.3.3.17027 22110782 PMC3219173

[16] Videria IFS Moura DFL Magina S. Mechanisms regulating melanogenesis. An Bras Dermatol (2013) 88(1):76–83. 10.1590/S0365-05962013000100009 23539007 PMC3699939

[17] Kasumagic-Halilovic E Prohic A Begovic B Ovcina-Kurtovic N Association between vitiligo and thyroid autoimmunity. J Thyroid Res (2011) 2011:3 10.4061/2011/938257 PMC3121018 21747969

[18] Yang L Beal MF. Determination of neurotransmitter levels in models of Parkinson’s disease by HPLC-ECD. Methods Mol Biol (2011) 793:401–15. 10.1007/978-1-61779-328-8_27 21913116

[19] Mohammed GF Gomaa AHA Al-Dhubaibi MS. Highlights in pathogenesis of vitiligo. World J Clin Cases (2015) 3(3):221–30. 10.12998/wjcc.v3.i3.221 25789295 PMC4360494

[20] Schallreuter KU Epidermal adrenergic signal transduction as part of the neuronal network in the human epidermis. Dermatol Symp Proc (1997) 2:37–40. 10.1038/jidsymp.1997.9 9487014

[21] Slominski AMD Zmijewski M Pawelek J L-tyrosine and L-DOPA as hormone-like regulators of melanocytes functions. Pigment Cell Melanoma Res (2012) 25(1):14–27. 10.1111/j.1755-148X.2011.00898.x 21834848 PMC3242935

[22] Cucchi ML Frattini P Santagostino G Preda S Orecchia G Catecholamines increases in the urine of non-segmental vitiligo especially during its active phase. Pigment Cell Res (2003) 16:111 10.1034/j.1600-0749.2003.00015.x 12622787

[23] Schallreuter KU Elwary SMA Gibbons NCJ Rokos H Wood JM. Activation/deactivation of acetylcholinesterase by H_2_O_2_: more evidence for oxidative stress in vitiligo. Biochem Biophys Res Commun (2004) 315:502–8. 10.1016/j.bbrc.2004.01.082 14766237

[24] Laddha NC Dwivedi M Mansuri MS Gani AR Ansarullah M Ramachandran AV Vitiligo: interplay between oxidative stress and immune system. Exp Dermatol (2013) 22(4):245–50. 10.1111/exd.12103 23425123

[25] Denat L Kadekaro AL Marrot L Leachman SA Abdel-Malek ZA. Melanocytes as instigators and victims of oxidative stress. J Invest Dermatol (2014) 134:1512–8. 10.1038/jid.2014.65 24573173 PMC4418514

[26] Park ES Kim SY Na JI Ryu HS Youn SW Kim DS Glutathione prevented dopamine-induced apoptosis of melanocytes and its signaling. J Dermatol Sci (2007) 47(2):141–9. 10.1016/j.jdermsci.2007.03.009 17481858

[27] Orecchia G Frattini P Cucchi ML Santagostino G Normal range plasma catecholamines in patients with generalized and acrofacial vitiligo: preliminary report. Dermatology (1994) 189:350–3. 10.1159/000246877 7873818

[28] Slominski A Wortsman J Tobin DJ. The cutaneous serotoninergic/melatoninergic system: securing a place under the sun. FASEB J (2005) 19:176–94. 10.1096/fj.04-2079rev 15677341

[29] Mosher DB Fitzpatrick TB Ortonne JP Hori Y Disorders of melanocytes. In: Fitzpatrick TB Eisen AZ Wolff K Freedberg IM Austen KF, editors. Dermatology in General Medicine. New York: McGraw Hill (1987). p. 810–8.

[30] Weatherhead B Logan A Interaction of a-melanocyte stimulating hormone, melatonin, cyclic AMP and cyclic GMP in the control of melanogenesis in hair follicle melanocytes in vitro. J Endocrinol (1981) 90:89–96. 10.1677/joe.0.0900089 6267154

[31] Herane MI Vitiligo and leukoderma in children. Clin Dermatol (2003) 21:283–95. 10.1016/S0738-081X(03)00048-8 14572699

[32] Chakraborty DP Roy S Chakraborty AK Vitiligo, psoralen, and melanogenesis – some observations and understanding. Pigment Cell Res (1996) 9:107–16. 10.1111/j.1600-0749.1996.tb00098.x 8888309

[33] Slominski A Tobin DJ Shibahara S Wortsman J. Melanin pigmentation in mammalian skin and its hormonal regulation. Physiol Rev (2004) 84:1155–228. 10.1152/physrev.00044.2003 15383650

[34] Kurbanov KH Berezov TT [Tryptophan metabolism in vitiligo]. Vopr Med Khim (1976) 22:683–7. 1014481

[35] Bystryn JC Serum antibodies in vitiligo patients. Clin Dermatol (1989) 7(2):136–45. 10.1016/0738-081X(89)90063-1 2667738

[36] Ongenea K Gel NV Naeyaert J. Evidence for an autoimmune pathogenesis of vitiligo. Pigment Cell Res (2003) 16:90–100. 10.1034/j.1600-0749.2003.00023.x 12622785

[37] Felsten LM Alikhan A Petronic-Rosic V. Vitiligo: a comprehensive overview part II: treatment options and approach to treatment. J Am Acad Dermatol (2011) 65:493–514. 10.1016/j.jaad.2010.10.043 21839316

[38] Langan EA Griffiths CE Paus R. Exploring the role of prolactin in psoriasis. Arch Dermatol Res (2012) 304:115–8. 10.1007/s00403-012-1208-6 22249743

[39] James WD Elston DM Berger TG Andrews GC Andrews’ diseases of the skin. In: James WD Berger TG Elston DM, editors. Clinical Dermatology. 11th ed London: Saunders Elsevier (2011). 959 p.

[40] Rokos H Beazley WD Schallreuter KU Oxidative stress in vitiligo: photo-oxidation of pterins produces H_2_O_2_ and pterin-6-carboxylic acid. Biochem Biophys Res Commun (2002) 292:805–11. 10.1006/bbrc.2002.6727 11944885

[41] Anderson D Schmid TE Baumgartner A Cemeli-Carratala E Brinkworth MH Wood JM. Oestrogenic compounds and oxidative stress (in human sperm and lymphocytes in the comet assay). Mutat Res (2003) 544:173–8. 10.1016/j.mrrev.2003.06.016 14644319

[42] Sheth VM Pandya AG. Melasma: a comprehensive update: part II. J Am Acad Dermatol (2011) 65(4):699–714. 10.1016/j.jaad.2011.06.001 21920242

[43] Natale CA Duperret EK Zhang J Sadeghi R Dahal A O’Brien KT Sex steroids regulate skin pigmentation through nonclassical membrane-bound receptors. Elife (2016) 5:e15104. 10.7554/eLife.15104 27115344 PMC4863824

[44] Kang HU Ortonne JP. What should be considered in treatment of melasma. Ann Dermatol (2010) 22(4):373–8. 10.5021/ad.2010.22.4.373 21165205 PMC2991712

[45] Kumar P Kumar N Thakur DS Patidar A Male hypogonadism: symptoms and treatment. J Adv Pharm Technol Res (2010) 1(3):297–301. 10.4103/0110-5558.72420 22247861 PMC3255409

[46] Kanwar AJ Kumaran MS. Childhood vitiligo: treatment paradigms. Indian J Dermatol (2012) 57:466–74. 10.4103/0019-5154.103067 23248365 PMC3519254

[47] Sukan M Maner F. The problems in sexual functions of vitiligo and chronic urticaria patients. J Sex Marital Ther (2007) 33:55–64. 10.1080/00926230600998482 17162488

[48] Sandoval-Cruz M Garcia-Carrasco M Sanchez-Porras R Mendoza-Pinto C Jimenez-Hernandez M Munguia-Realpozo P Immunopathogenesis of vitiligo. Autoimmun Rev (2011) 10:762–5. 10.1016/j.autrev.2011.02.004 21334464

[49] Colucci R Dragoni F Moretti S. Oxidative stress and immune system in vitiligo and thyroid diseases. Oxid Med Cell Longev (2015) 2015:7. 10.1155/2015/631927 25838868 PMC4370195

[50] Jishna P Binitha MP Abdul Latheef EN Anilakumari VP Prevalence of thyroid dysfunction and anti-thyroid peroxidase antibodies in vitiligo patients. Int J Res Dermatol (2017) 3:140–4. 10.18203/issn.2455-4529.IntJResDermatol20170803

[51] Bahrami Z Hedayati M Taghikhani M Azizi F. Effect of testosterone on thyroid weight and function in iodine deficient castrated rats. Horm Metab Res (2009) 41(10):762–6. 10.1055/s-0029-1225629 19585407

[52] Biswas M Chattopadhyay A Mridha K Biswas T Biswas J Kamal Hassan Sk A study on association between vitiligo and thyroid dysfunction. J Dental Med Sci (2015) 14(10):34–7. 10.9790/0853-141093437

[53] Prindaville B Rivkees SA. Incidence of vitiligo in children with Graves’ disease and Hashimoto’s thyroiditis. Int J Pediatr Endocrinol (2011) 2011(1):18. 10.1186/1687-9856-2011-18 22099887 PMC3256118

[54] Rousseau K Kauser S Pritchard LE Warhurst A Oliver RL Slominski A Proopiomelanocortin (POMC), the ACTH/melanocortin precursor, is secreted by human epidermal keratinocytes and melanocytes and stimulates melanogenesis. FASEB J (2007) 21(8):1844–56. 10.1096/fj.06-7398com 17317724 PMC2253185

[55] Yamamoto H Yamane T Iguchi K Tanaka K Iddamalgoda A Unno K Melanin production through novel processing of proopiomelanocortin in the extracellular compartment of the auricular skin of C57BL/6 mice after UV-irradiation. Sci Rep (2015) 5:14579. 10.1038/srep14579 26417724 PMC4586518

[56] Rodríguez CI Setaluri V Cyclic AMP (camp) signaling in melanocytes and melanoma. Arch Biochem Biophys (2014) 563:22–7. 10.1016/j.abb.2014.07.003 25017568

[57] Slominski A Zbytek B Szczesniewski A Semak I Kaminski J Sweatman T CRH stimulation of corticosteroids production in melanocytes is mediated by ACTH. Am J Physiol Endocrinol Metab (2005) 288:E701–6. 10.1152/ajpendo.00519.2004 15572653

[58] Zbytek B Pfeffer LM Slominski AT Corticotropin releasing hormone stimulates NF-kappa B in human epidermal keratinocytes. J Endocrinol (2004) 181:R1–7. 10.1677/joe.0.181R001 15171702 PMC1262682

[59] Zbytek B Pfeffer LM Slominski AT CRH inhibits NF-kB signaling in human melanocytes. Peptides (2006) 27:3276–83. 10.1016/j.peptides.2006.07.017 16959375 PMC1839005

[60] Morrone A Picardo M de Luca C Terminali O Passi S Ippolito F Catecholamines and vitiligo. Pigment Cell Res (1992) 5:65–9. 10.1111/j.1600-0749.1992.tb00003.x 1321419

[61] Taub DD Neuroendocrine interactions in the immune system. Cell Immunol (2008) 252(1–2):1–6. 10.1016/j.cellimm.2008.05.006 18619587 PMC2562609

[62] Chen Y Johnson AG. In vivo activation of macrophages by prolactin from young and aging mice. Int J Immunopharmacol (1993) 15(1):39–45. 10.1016/0192-0561(93)90029-X 8381775

