# Supplementary material for: Neural and Endocrinal Pathobiochemistry of Vitiligo: Comparative Study for a Hypothesized Mechanism
Source: Front Endocrinol (Lausanne). 2018 Apr 25;9:197. doi: 10.3389/fendo.2018.00197 (PMC5996913; doi:10.3389/fendo.2018.00197)
Supplement: Supplementary file 1 [file data_sheet_1.PDF]

Individual Data Sheet 1

Note that; CM = control male; CF = control female; SM = stable vitiligo male; SF = stable vitiligo female; AM = active vitiligo male; AF = active vitiligo female; H = hormone or studied parameter.

**ACTH (**pmol/L**) (Age, Sex, & BMI)**																		
	**CM**			**CF**			**SM**			**SF**			**AM**			**AF**		
	**Age**	**BMI**	**H**	**Age**	**BMI**	**H**	**Age**	**BMI**	**H**	**Age**	**BMI**	**H**	**Age**	**BMI**	**H**	**Age**	**BMI**	**H**
**1**	18.	18.	8.0	20.	22.	5.0	23.	25.	4.5	22.	24.	2.5	21.	22.	2.8	22.	23.	1.5
**2**	18.	18.	8.0	21.	23.	5.0	21.	25.	4.5	22.	25.	2.5	21.	22.	2.8	22.	26.	1.5
**3**	19.	22.	8.0	20.	23.	5.5	23.	26.	5.0	22.	25.	2.0	21.	23.	2.9	22.	26.	1.4
**4**	19.	23.	8.0	20.	22.	5.5	35.	30.	5.0	23.	23.	2.0	22.	22.	2.9	22.	27.	1.4
**5**	18.	21.	9.0	20.	22.	6.0	35.	29.	5.5	22.	23.	2.2	21.	23.	3.0	23.	23.	1.3
**6**	40.	23.	9.0	21.	29.	6.0	35.	29.	5.5	44.	26.	2.2	34.	23.	3.0	22.	24.	1.3
**7**	40.	18.	9.0	21.	22.	6.5	45.	21.	4.0	44.	24.	2.3	34.	22.	3.0	22.	24.	1.2
**8**	41.	24.	7.0	21.	18.	6.5	45.	23.	4.0	44.	25.	2.3	34.	22.	2.8	44.	23.	1.2
**9**	41.	21.	7.0	23.	17.	7.0	46.	22.	4.3	44.	25.	2.6	35.	21.	2.7	44.	22.	1.6
**10**	42.	22.	6.0	23.	28.	7.0	44.	23.	4.2	44.	23.	2.7	46.	21.	2.7	44.	24.	1.6
**11**	34.	17.	8.0	44.	23.	4.5				36.	23.	2.8	46.	20.	2.6	43.	25.	1.7
**12**	33.	27.	7.5	44.	23.	4.5				33.	20.	2.5	45.	21.	2.6	40.	26.	1.7
**13**	33.	20.	8.5	43.	26.	4.0				33.	22.	2.6	46.	20.	2.7	35.	24.	1.8
**14**	33.	21.	8.0	43.	26.	4.0				40.	23.	2.7	46.	23.	2.9	35.	25.	1.8
**15**	32.	22.	7.9	44.	27.	3.5				29.	21.	2.5	45.	23.	3.0	33.	24.	1.9
**16**				42.	21.	3.5										32.	24.	2.0
**17**				42.	20.	3.0										31.	23.	1.4
**18**				44.	22.	3.0										34.	24.	1.4
**19**				42.	17.	5.0										33.	28.	1.5
**20**				41.	24.	5.2										32.	29.	1.5
**21**				33.	25.	5.2												
**22**				33.	26.	5.0												
**23**				31.	22.	4.9												
**24**				32.	16.	4.8												
**25**				33.	23.	5.0												

Individual Data Sheet 2

Note that; CM = control male; CF = control female; SM = stable vitiligo male; SF = stable vitiligo female; AM = active vitiligo male; AF = active vitiligo female; H = hormone or studied parameter.

Cortisol (nmol/L) (Age, Sex, & BMI)																		
	CM			CF			SM			SF			AM			AF		
	Age	BMI	H	Age	BMI	H	Age	BMI	H	Age	BMI	H	Age	BMI	H	Age	BMI	H
1	18.	18.	250.0	20.	22.	450.0	23.	25.	300.0	22.	24.	470.0	21.	22.	710.0	22.	23.	860.0
2	18.	18.	255.0	21.	23.	455.0	21.	25.	310.0	22.	25.	470.0	21.	22.	720.0	22.	26.	860.0
3	19.	22.	255.0	20.	23.	455.0	23.	26.	310.0	22.	25.	480.0	21.	23.	720.0	22.	26.	890.0
4	19.	23.	251.0	20.	22.	460.0	35.	30.	320.0	23.	23.	480.0	22.	22.	730.0	22.	27.	890.0
5	18.	21.	251.0	20.	22.	460.0	35.	29.	320.0	22.	23.	490.0	21.	23.	730.0	23.	23.	900.0
6	40.	23.	252.0	21.	29.	465.0	35.	29.	330.0	44.	26.	490.0	34.	23.	750.0	22.	24.	900.0
7	40.	18.	252.0	21.	22.	465.0	45.	21.	330.0	44.	24.	495.0	34.	22.	750.0	22.	24.	850.0
8	41.	24.	245.0	21.	18.	470.0	45.	23.	300.0	44.	25.	495.0	34.	22.	700.0	44.	23.	850.0
9	41.	21.	245.0	23.	17.	470.0	46.	22.	300.0	44.	25.	460.0	35.	21.	700.0	44.	22.	840.0
10	42.	22.	246.0	23.	28.	440.0	44.	23.	290.0	44.	23.	460.0	46.	21.	700.0	44.	24.	840.0
11	34.	17.	246.0	44.	23.	440.0				36.	23.	455.0	46.	20.	690.0	43.	25.	850.0
12	33.	27.	252.0	44.	23.	435.0				33.	20.	455.0	45.	21.	690.0	40.	26.	850.0
13	33.	20.	254.0	43.	26.	435.0				33.	22.	450.0	46.	20.	680.0	35.	24.	880.0
14	33.	21.	252.0	43.	26.	436.0				40.	23.	475.0	46.	23.	680.0	35.	25.	880.0
15	32.	22.	250.0	44.	27.	450.0				29.	21.	470.0	45.	23.	705.0	33.	24.	890.0
16				42.	21.	449.0										32.	24.	870.0
17				42.	20.	446.0										31.	23.	830.0
18				44.	22.	452.0										34.	24.	855.0
19				42.	17.	457.0										33.	28.	860.0
20				41.	24.	460.0										32.	29.	870.0
21				33.	25.	455.0												
22				33.	26.	460.0												
23				31.	22.	455.0												
24				32.	16.	456.0												
25				33.	23.	480.0												

Individual Data Sheet 3

Note that; CM = control male; CF = control female; SM = stable vitiligo male; SF = stable vitiligo female; AM = active vitiligo male; AF = active vitiligo female; H = hormone or studied parameter.

Epinephrine ((pmol/L) (Age, Sex, & BMI)																		
	**CM**			**CF**			**SM**			**SF**			**AM**			**AF**		
	**Age**	**BMI**	**H**	**Age**	**BMI**	**H**	**Age**	**BMI**	**H**	**Age**	**BMI**	**H**	**Age**	**BMI**	**H**	**Age**	**BMI**	**H**
**1**	18.	18.	350.	20.	22.	300.	23.	25.	400.	22.	24.	380.	21.	22.	650.	22.	23.	590.
**2**	18.	18.	360.	21.	23.	305.	21.	25.	405.	22.	25.	370.	21.	22.	660.	22.	26.	550.
**3**	19.	22.	340.	20.	23.	295.	23.	26.	410.	22.	25.	375.	21.	23.	640.	22.	26.	555.
**4**	19.	23.	370.	20.	22.	310.	35.	30.	415.	23.	23.	377.	22.	22.	645.	22.	27.	560.
**5**	18.	21.	320.	20.	22.	290.	35.	29.	390.	22.	23.	380.	21.	23.	655.	23.	23.	565.
**6**	40.	23.	355.	21.	29.	295.	35.	29.	395.	44.	26.	382.	34.	23.	640.	22.	24.	577.
**7**	40.	18.	365.	21.	22.	307.	45.	21.	385.	44.	24.	385.	34.	22.	644.	22.	24.	570.
**8**	41.	24.	345.	21.	18.	302.	45.	23.	380.	44.	25.	390.	34.	22.	650.	44.	23.	600.
**9**	41.	21.	350.	23.	17.	292.	46.	22.	382.	44.	25.	375.	35.	21.	651.	44.	22.	610.
**10**	42.	22.	347.	23.	28.	303.	44.	23.	400.	44.	23.	372.	46.	21.	649.	44.	24.	605.
**11**	34.	17.	352.	44.	23.	312.				36.	23.	381.	46.	20.	630.	43.	25.	607.
**12**	33.	27.	330.	44.	23.	285.				33.	20.	382.	45.	21.	670.	40.	26.	595.
**13**	33.	20.	366.	43.	26.	288.				33.	22.	379.	46.	20.	655.	35.	24.	599.
**14**	33.	21.	342.	43.	26.	292.				40.	23.	377.	46.	23.	625.	35.	25.	570.
**15**	32.	22.	358.	44.	27.	301.				29.	21.	383.	45.	23.	651.	33.	24.	620.
**16**				42.	21.	305.										32.	24.	611.
**17**				42.	20.	308.										31.	23.	610.
**18**				44.	22.	320.										34.	24.	585.
**19**				42.	17.	325.										33.	28.	592.
**20**				41.	24.	291.										32.	29.	588.
**21**				33.	25.	290.												
**22**				33.	26.	299.												
**23**				31.	22.	319.												
**24**				32.	16.	300.												
**25**				33.	23.	305.												

Individual Data Sheet 4

Note that; CM = control male; CF = control female; SM = stable vitiligo male; SF = stable vitiligo female; AM = active vitiligo male; AF = active vitiligo female; H = hormone or studied parameter.

**Nor-Epinephrine** (nmol/L) **(Age, Sex, & BMI)**																		
	**CM**			**CF**			**SM**			**SF**			**AM**			**AF**		
	**Age**	**BMI**	**H**	**Age**	**BMI**	**H**	**Age**	**BMI**	**H**	**Age**	**BMI**	**H**	**Age**	**BMI**	**H**	**Age**	**BMI**	**H**
**1**	18.	18.	2222.	20.	22.	1950.	23.	25.	2500.	22.	24.	2350.	21.	22.	3100.	22.	23.	2760.
**2**	18.	18.	2200.	21.	23.	1900.	21.	25.	2400.	22.	25.	2300.	21.	22.	3000.	22.	26.	2700.
**3**	19.	22.	2100.	20.	23.	2000.	23.	26.	2300.	22.	25.	2200.	21.	23.	2900.	22.	26.	2740.
**4**	19.	23.	2150.	20.	22.	1980.	35.	30.	2250.	23.	23.	2250.	22.	22.	2990.	22.	27.	2700.
**5**	18.	21.	2200.	20.	22.	1970.	35.	29.	2350.	22.	23.	2240.	21.	23.	3050.	23.	23.	2660.
**6**	40.	23.	2220.	21.	29.	1850.	35.	29.	2440.	44.	26.	2300.	34.	23.	3000.	22.	24.	2670.
**7**	40.	18.	2300.	21.	22.	1880.	45.	21.	2400.	44.	24.	2350.	34.	22.	2850.	22.	24.	2690.
**8**	41.	24.	2000.	21.	18.	1890.	45.	23.	2250.	44.	25.	2340.	34.	22.	2950.	44.	23.	2688.
**9**	41.	21.	1999.	23.	17.	1900.	46.	22.	2550.	44.	25.	2310.	35.	21.	3200.	44.	22.	2950.
**10**	42.	22.	2050.	23.	28.	1910.	44.	23.	2410.	44.	23.	2200.	46.	21.	3144.	44.	24.	2701.
**11**	34.	17.	2200.	44.	23.	1920.				36.	23.	2290.	46.	20.	3000.	43.	25.	2710.
**12**	33.	27.	2210.	44.	23.	1855.				33.	20.	2280.	45.	21.	2904.	40.	26.	2730.
**13**	33.	20.	2100.	43.	26.	1890.				33.	22.	2295.	46.	20.	2999.	35.	24.	2800.
**14**	33.	21.	2155.	43.	26.	1750.				40.	23.	2301.	46.	23.	3005.	35.	25.	2650.
**15**	32.	22.	2250.	44.	27.	1670.				29.	21.	2307.	45.	23.	3010.	33.	24.	2700.
**16**				42.	21.	2000.										32.	24.	2712.
**17**				42.	20.	2100.										31.	23.	2780.
**18**				44.	22.	2025.										34.	24.	2670.
**19**				42.	17.	2150.										33.	28.	2600.
**20**				41.	24.	1700.										32.	29.	2750.
**21**				33.	25.	1990.												
**22**				33.	26.	1900.												
**23**				31.	22.	1980.												
**24**				32.	16.	1899.												
**25**				33.	23.	1950.												

Individual Data Sheet 5

Note that; CM = control male; CF = control female; SM = stable vitiligo male; SF = stable vitiligo female; AM = active vitiligo male; AF = active vitiligo female; H = hormone or studied parameter.

**Dopamine** (pmol/L) **(Age, Sex, & BMI)**																		
	**CM**			**CF**			**SM**			**SF**			**AM**			**AF**		
	**Age**	**BMI**	**H**	**Age**	**BMI**	**H**	**Age**	**BMI**	**H**	**Age**	**BMI**	**H**	**Age**	**BMI**	**H**	**Age**	**BMI**	**H**
**1**	18.	18.	210.	20.	22.	180.	23.	25.	530.	22.	24.	490.	21.	22.	570.	22.	23.	510.
**2**	18.	18.	200.	21.	23.	185.	21.	25.	535.	22.	25.	500.	21.	22.	580.	22.	26.	520.
**3**	19.	22.	205.	20.	23.	180.	23.	26.	540.	22.	25.	501.	21.	23.	585.	22.	26.	530.
**4**	19.	23.	202.	20.	22.	182.	35.	30.	544.	23.	23.	510.	22.	22.	575.	22.	27.	533.
**5**	18.	21.	215.	20.	22.	184.	35.	29.	550.	22.	23.	515.	21.	23.	570.	23.	23.	500.
**6**	40.	23.	220.	21.	29.	190.	35.	29.	510.	44.	26.	450.	34.	23.	560.	22.	24.	490.
**7**	40.	18.	222.	21.	22.	170.	45.	21.	520.	44.	24.	455.	34.	22.	580.	22.	24.	499.
**8**	41.	24.	209.	21.	18.	175.	45.	23.	529.	44.	25.	460.	34.	22.	566.	44.	23.	495.
**9**	41.	21.	208.	23.	17.	177.	46.	22.	511.	44.	25.	465.	35.	21.	565.	44.	22.	505.
**10**	42.	22.	200.	23.	28.	181.	44.	23.	512.	44.	23.	560.	46.	21.	568.	44.	24.	510.
**11**	34.	17.	211.	44.	23.	180.				36.	23.	555.	46.	20.	572.	43.	25.	512.
**12**	33.	27.	213.	44.	23.	192.				33.	20.	480.	45.	21.	540.	40.	26.	500.
**13**	33.	20.	199.	43.	26.	170.				33.	22.	495.	46.	20.	590.	35.	24.	456.
**14**	33.	21.	195.	43.	26.	173.				40.	23.	500.	46.	23.	567.	35.	25.	470.
**15**	32.	22.	220.	44.	27.	160.				29.	21.	505.	45.	23.	570.	33.	24.	550.
**16**				42.	21.	166.										32.	24.	515.
**17**				42.	20.	175.										31.	23.	488.
**18**				44.	22.	178.										34.	24.	499.
**19**				42.	17.	200.										33.	28.	500.
**20**				41.	24.	184.										32.	29.	504.
**21**				33.	25.	181.												
**22**				33.	26.	186.												
**23**				31.	22.	179.												
**24**				32.	16.	175.												
**25**				33.	23.	188.												

Individual Data Sheet 6

Note that; CM = control male; CF = control female; SM = stable vitiligo male; SF = stable vitiligo female; AM = active vitiligo male; AF = active vitiligo female; H = hormone or studied parameter.

HVA (ng/ml) (Age, Sex, & BMI)																		
	**CM**			**CF**			**SM**			**SF**			**AM**			**AF**		
	**Age**	**BMI**	**H**	**Age**	**BMI**	**H**	**Age**	**BMI**	**H**	**Age**	**BMI**	**H**	**Age**	**BMI**	**H**	**Age**	**BMI**	**H**
**1**	18.	18.	10.	20.	22.	8.	23.	25.	25.	22.	24.	22.	21.	22.	30.	22.	23.	25.
**2**	18.	18.	11.	21.	23.	8.	21.	25.	24.	22.	25.	23.	21.	22.	31.	22.	26.	24.
**3**	19.	22.	12.	20.	23.	7.	23.	26.	24.	22.	25.	24.	21.	23.	32.	22.	26.	23.
**4**	19.	23.	9.	20.	22.	9.	35.	30.	23.	23.	23.	25.	22.	22.	33.	22.	27.	24.
**5**	18.	21.	8.	20.	22.	7.	35.	29.	23.	22.	23.	20.	21.	23.	34.	23.	23.	23.
**6**	40.	23.	10.	21.	29.	9.	35.	29.	22.	44.	26.	20.	34.	23.	35.	22.	24.	22.
**7**	40.	18.	10.	21.	22.	9.	45.	21.	21.	44.	24.	21.	34.	22.	30.	22.	24.	26.
**8**	41.	24.	11.	21.	18.	8.	45.	23.	26.	44.	25.	22.	34.	22.	29.	44.	23.	30.
**9**	41.	21.	12.	23.	17.	8.	46.	22.	26.	44.	25.	27.	35.	21.	28.	44.	22.	32.
**10**	42.	22.	8.	23.	28.	7.	44.	23.	27.	44.	23.	19.	46.	21.	26.	44.	24.	24.
**11**	34.	17.	8.	44.	23.	7.				36.	23.	20.	46.	20.	25.	43.	25.	25.
**12**	33.	27.	7.	44.	23.	6.				33.	20.	20.	45.	21.	23.	40.	26.	26.
**13**	33.	20.	10.	43.	26.	6.				33.	22.	22.	46.	20.	36.	35.	24.	20.
**14**	33.	21.	11.	43.	26.	9.				40.	23.	23.	46.	23.	37.	35.	25.	35.
**15**	32.	22.	9.	44.	27.	9.				29.	21.	21.	45.	23.	40.	33.	24.	34.
**16**				42.	21.	10.										32.	24.	19.
**17**				42.	20.	6.										31.	23.	18.
**18**				44.	22.	6.										34.	24.	22.
**19**				42.	17.	8.										33.	28.	25.
**20**				41.	24.	8.										32.	29.	24.
**21**				33.	25.	9.												
**22**				33.	26.	6.												
**23**				31.	22.	11.												
**24**				32.	16.	8.												
**25**				33.	23.	9.												

Individual Data Sheet 7

Note that; CM = control male; CF = control female; SM = stable vitiligo male; SF = stable vitiligo female; AM = active vitiligo male; AF = active vitiligo female; H = hormone or studied parameter.

Serotonin (ng/ml) (Age, Sex, & BMI)																		
	CM			CF			SM			SF			AM			AF		
	Age	BMI	H	Age	BMI	H	Age	BMI	H	Age	BMI	H	Age	BMI	H	Age	BMI	H
**1**	18.	18.	25.	20.	22.	35.	23.	25.	55.	22.	24.	65.	21.	22.	75.	22.	23.	83.
**2**	18.	18.	26.	21.	23.	36.	21.	25.	54.	22.	25.	66.	21.	22.	74.	22.	26.	82.
**3**	19.	22.	27.	20.	23.	37.	23.	26.	53.	22.	25.	67.	21.	23.	72.	22.	26.	81.
**4**	19.	23.	28.	20.	22.	38.	35.	30.	52.	23.	23.	68.	22.	22.	70.	22.	27.	80.
**5**	18.	21.	29.	20.	22.	39.	35.	29.	51.	22.	23.	69.	21.	23.	65.	23.	23.	79.
**6**	40.	23.	30.	21.	29.	40.	35.	29.	50.	44.	26.	70.	34.	23.	66.	22.	24.	77.
**7**	40.	18.	24.	21.	22.	44.	45.	21.	57.	44.	24.	72.	34.	22.	67.	22.	24.	84.
**8**	41.	24.	23.	21.	18.	33.	45.	23.	58.	44.	25.	64.	34.	22.	68.	44.	23.	85.
**9**	41.	21.	23.	23.	17.	32.	46.	22.	59.	44.	25.	63.	35.	21.	77.	44.	22.	87.
**10**	42.	22.	22.	23.	28.	32.	44.	23.	62.	44.	23.	62.	46.	21.	78.	44.	24.	89.
**11**	34.	17.	21.	44.	23.	33.				36.	23.	61.	46.	20.	79.	43.	25.	90.
**12**	33.	27.	20.	44.	23.	31.				33.	20.	60.	45.	21.	80.	40.	26.	95.
**13**	33.	20.	25.	43.	26.	30.				33.	22.	64.	46.	20.	81.	35.	24.	80.
**14**	33.	21.	26.	43.	26.	34.				40.	23.	63.	46.	23.	82.	35.	25.	75.
**15**	32.	22.	25.	44.	27.	35.				29.	21.	60.	45.	23.	84.	33.	24.	77.
**16**				42.	21.	32.										32.	24.	89.
**17**				42.	20.	37.										31.	23.	90.
**18**				44.	22.	36.										34.	24.	83.
**19**				42.	17.	31.										33.	28.	82.
**20**				41.	24.	30.										32.	29.	85.
**21**				33.	25.	35.												
**22**				33.	26.	36.												
**23**				31.	22.	29.												
**24**				32.	16.	29.												
**25**				33.	23.	30.												

Individual Data Sheet 8

Note that; CM = control male; CF = control female; SM = stable vitiligo male; SF = stable vitiligo female; AM = active vitiligo male; AF = active vitiligo female; H = hormone or studied parameter.

HIAA (ng/ml) (Age, Sex, & BMI)																		
	CM			CF			SM			SF			AM			AF		
	Age	BMI	H	Age	BMI	H	Age	BMI	H	Age	BMI	H	Age	BMI	H	Age	BMI	H
**1**	18.	18.	6.	20.	22.	6.	23.	25.	8.	22.	24.	10.	21.	22.	11.	22.	23.	12.
**2**	18.	18.	6.	21.	23.	6.	21.	25.	8.	22.	25.	11.	21.	22.	12.	22.	26.	13.
**3**	19.	22.	5.	20.	23.	5.	23.	26.	9.	22.	25.	12.	21.	23.	13.	22.	26.	12.
**4**	19.	23.	5.	20.	22.	5.	35.	30.	9.	23.	23.	13.	22.	22.	14.	22.	27.	11.
**5**	18.	21.	7.	20.	22.	7.	35.	29.	10.	22.	23.	10.	21.	23.	11.	23.	23.	14.
**6**	40.	23.	7.	21.	29.	7.	35.	29.	11.	44.	26.	9.	34.	23.	10.	22.	24.	10.
**7**	40.	18.	8.	21.	22.	8.	45.	21.	9.	44.	24.	8.	34.	22.	9.	22.	24.	15.
**8**	41.	24.	8.	21.	18.	8.	45.	23.	7.	44.	25.	7.	34.	22.	8.	44.	23.	12.
**9**	41.	21.	4.	23.	17.	4.	46.	22.	6.	44.	25.	6.	35.	21.	7.	44.	22.	11.
**10**	42.	22.	4.	23.	28.	4.	44.	23.	6.	44.	23.	10.	46.	21.	11.	44.	24.	13.
**11**	34.	17.	4.	44.	23.	4.				36.	23.	11.	46.	20.	12.	43.	25.	12.
**12**	33.	27.	6.	44.	23.	6.				33.	20.	12.	45.	21.	13.	40.	26.	11.
**13**	33.	20.	6.	43.	26.	6.				33.	22.	10.	46.	20.	9.	35.	24.	10.
**14**	33.	21.	5.	43.	26.	5.				40.	23.	9.	46.	23.	10.	35.	25.	14.
**15**	32.	22.	5.	44.	27.	5.				29.	21.	9.	45.	23.	9.	33.	24.	14.
**16**				42.	21.	7.										32.	24.	12.
**17**				42.	20.	7.										31.	23.	11.
**18**				44.	22.	8.										34.	24.	13.
**19**				42.	17.	8.										33.	28.	14.
**20**				41.	24.	7.										32.	29.	12.
**21**				33.	25.	7.												
**22**				33.	26.	5.												
**23**				31.	22.	5.												
**24**				32.	16.	5.												
**25**				33.	23.	5.												

Individual Data Sheet 9

Note that; CM = control male; CF = control female; SM = stable vitiligo male; SF = stable vitiligo female; AM = active vitiligo male; AF = active vitiligo female; H = hormone or studied parameter.

Melatonin (ng/ml) (Age, Sex, & BMI)																		
	CM			CF			SM			SF			AM			AF		
	Age	BMI	H	Age	BMI	H	Age	BMI	H	Age	BMI	H	Age	BMI	H	Age	BMI	H
**1**	18.	18.	1.5	20.	22.	2.5	23.	25.	5.	22.	24.	7.	21.	22.	7.	22.	23.	9.
**2**	18.	18.	1.1	21.	23.	2.6	21.	25.	4.	22.	25.	6.	21.	22.	7.	22.	26.	9.
**3**	19.	22.	1.3	20.	23.	2.7	23.	26.	4.	22.	25.	6.	21.	23.	7.	22.	26.	9.
**4**	19.	23.	1.4	20.	22.	2.8	35.	30.	5.	23.	23.	5.	22.	22.	8.	22.	27.	8.
**5**	18.	21.	1.6	20.	22.	2.9	35.	29.	5.	22.	23.	5.	21.	23.	8.	23.	23.	8.
**6**	40.	23.	1.7	21.	29.	3.0	35.	29.	5.	44.	26.	7.	34.	23.	8.	22.	24.	10.
**7**	40.	18.	1.8	21.	22.	2.4	45.	21.	6.	44.	24.	7.	34.	22.	6.	22.	24.	10.
**8**	41.	24.	1.9	21.	18.	2.3	45.	23.	6.	44.	25.	8.	34.	22.	6.	44.	23.	7.
**9**	41.	21.	2.0	23.	17.	2.2	46.	22.	4.	44.	25.	8.	35.	21.	6.	44.	22.	7.
**10**	42.	22.	1.0	23.	28.	2.1	44.	23.	5.	44.	23.	9.	46.	21.	9.	44.	24.	11.
**11**	34.	17.	0.9	44.	23.	2.0				36.	23.	9.	46.	20.	9.	43.	25.	11.
**12**	33.	27.	1.4	44.	23.	2.5				33.	20.	10.	45.	21.	5.	40.	26.	12.
**13**	33.	20.	1.5	43.	26.	2.4				33.	22.	4.	46.	20.	5.	35.	24.	12.
**14**	33.	21.	1.6	43.	26.	2.7				40.	23.	5.	46.	23.	7.	35.	25.	6.
**15**	32.	22.	1.3	44.	27.	2.3				29.	21.	7.	45.	23.	7.	33.	24.	6.
**16**				42.	21.	2.2										32.	24.	6.
**17**				42.	20.	2.1										31.	23.	6.
**18**				44.	22.	2.5										34.	24.	9.
**19**				42.	17.	2.6										33.	28.	9.
**20**				41.	24.	2.5										32.	29.	8.
**21**				33.	25.	2.4												
**22**				33.	26.	2.6												
**23**				31.	22.	2.7												
**24**				32.	16.	2.2												
**25**				33.	23.	2.5												

Individual Data Sheet 10

Note that; CM = control male; CF = control female; SM = stable vitiligo male; SF = stable vitiligo female; AM = active vitiligo male; AF = active vitiligo female; H = hormone or studied parameter.

Testosterone (nmol/L)(Age, Sex, & BMI)

	**CM**			**CF**			**SM**			**SF**			**AM**			**AF**		
	**Age**	**BMI**	**H**	**Age**	**BMI**	**H**	**Age**	**BMI**	**H**	**Age**	**BMI**	**H**	**Age**	**BMI**	**H**	**Age**	**BMI**	**H**
**1**	18.	18.	25.0	20.	22.	1.5	23.	25.	10.0	22.	24.	2.0	21.	22.	2.5	22.	23.	1.0
**2**	18.	18.	26.0	21.	23.	1.6	21.	25.	10.0	22.	25.	2.0	21.	22.	2.5	22.	26.	0.9
**3**	19.	22.	26.0	20.	23.	1.6	23.	26.	11.0	22.	25.	3.0	21.	23.	2.5	22.	26.	0.9
**4**	19.	23.	27.0	20.	22.	1.7	35.	30.	11.0	23.	23.	3.0	22.	22.	2.4	22.	27.	0.9
**5**	18.	21.	27.0	20.	22.	1.7	35.	29.	12.0	22.	23.	3.0	21.	23.	2.4	23.	23.	0.8
**6**	40.	23.	30.0	21.	29.	1.8	35.	29.	12.0	44.	26.	2.0	34.	23.	2.4	22.	24.	0.8
**7**	40.	18.	31.0	21.	22.	1.8	45.	21.	9.0	44.	24.	2.0	34.	22.	2.6	22.	24.	1.1
**8**	41.	24.	24.0	21.	18.	1.9	45.	23.	9.0	44.	25.	1.0	34.	22.	2.6	44.	23.	1.1
**9**	41.	21.	24.0	23.	17.	1.9	46.	22.	8.0	44.	25.	1.0	35.	21.	2.6	44.	22.	1.2
**10**	42.	22.	23.0	23.	28.	2.0	44.	23.	7.0	44.	23.	1.0	46.	21.	2.7	44.	24.	1.2
**11**	34.	17.	23.0	44.	23.	1.4				36.	23.	2.0	46.	20.	2.7	43.	25.	1.2
**12**	33.	27.	22.0	44.	23.	1.4				33.	20.	2.0	45.	21.	2.3	40.	26.	1.3
**13**	33.	20.	22.0	43.	26.	1.2				33.	22.	3.0	46.	20.	2.3	35.	24.	0.8
**14**	33.	21.	20.0	43.	26.	1.2				40.	23.	3.0	46.	23.	2.5	35.	25.	0.6
**15**	32.	22.	28.0	44.	27.	1.2				29.	21.	1.0	45.	23.	2.4	33.	24.	0.6
**16**				42.	21.	1.3										32.	24.	1.0
**17**				42.	20.	1.4										31.	23.	1.0
**18**				44.	22.	1.5										34.	24.	0.9
**19**				42.	17.	1.5										33.	28.	0.9
**20**				41.	24.	1.6										32.	29.	1.4
**21**				33.	25.	1.7												
**22**				33.	26.	1.4												
**23**				31.	22.	1.4												
**24**				32.	16.	1.5												
**25**				33.	23.	1.5												

Individual Data Sheet 11

Note that; CM = control male; CF = control female; SM = stable vitiligo male; SF = stable vitiligo female; AM = active vitiligo male; AF = active vitiligo female; H = hormone or studied parameter.

Estradiol (pmol/L) (Age, Sex, & BMI)																		
	CM			CF			SM			SF			AM			AF		
	Age	BMI	H	Age	BMI	H	Age	BMI	H	Age	BMI	H	Age	BMI	H	Age	BMI	H
1	18.	18.	110.0	20.	22.	1250.0	23.	25.	125.0	22.	24.	1300.0	21.	22.	165.0	22.	23.	2100.0
2	18.	18.	112.0	21.	23.	1250.0	21.	25.	125.0	22.	25.	1310.0	21.	22.	165.0	22.	26.	2150.0
3	19.	22.	113.0	20.	23.	1270.0	23.	26.	126.0	22.	25.	1320.0	21.	23.	166.0	22.	26.	2200.0
4	19.	23.	115.0	20.	22.	1270.0	35.	30.	126.0	23.	23.	1350.0	22.	22.	167.0	22.	27.	2300.0
5	18.	21.	100.0	20.	22.	1280.0	35.	29.	130.0	22.	23.	1290.0	21.	23.	168.0	23.	23.	2250.0
6	40.	23.	105.0	21.	29.	1280.0	35.	29.	130.0	44.	26.	1280.0	34.	23.	175.0	22.	24.	2050.0
7	40.	18.	99.0	21.	22.	1230.0	45.	21.	120.0	44.	24.	1270.0	34.	22.	160.0	22.	24.	2050.0
8	41.	24.	95.0	21.	18.	1230.0	45.	23.	120.0	44.	25.	1290.0	34.	22.	161.0	44.	23.	1950.0
9	41.	21.	99.0	23.	17.	1240.0	46.	22.	123.0	44.	25.	1300.0	35.	21.	160.0	44.	22.	1900.0
10	42.	22.	94.0	23.	28.	1240.0	44.	23.	123.0	44.	23.	1340.0	46.	21.	163.0	44.	24.	1990.0
11	34.	17.	114.0	44.	23.	1250.0				36.	23.	1260.0	46.	20.	160.0	43.	25.	2100.0
12	33.	27.	110.0	44.	23.	1300.0				33.	20.	1250.0	45.	21.	170.0	40.	26.	2100.0
13	33.	20.	107.0	43.	26.	1200.0				33.	22.	1360.0	46.	20.	166.0	35.	24.	2070.0
14	33.	21.	112.0	43.	26.	1280.0				40.	23.	1300.0	46.	23.	164.0	35.	25.	2090.0
15	32.	22.	116.0	44.	27.	1220.0				29.	21.	1310.0	45.	23.	166.0	33.	24.	2120.0
16				42.	21.	1255.0										32.	24.	2500.0
17				42.	20.	1255.0										31.	23.	1850.0
18				44.	22.	1245.0										34.	24.	1970.0
19				42.	17.	1245.0										33.	28.	2220.0
20				41.	24.	1240.0										32.	29.	2100.0
21				33.	25.	1260.0												
22				33.	26.	1230.0												
23				31.	22.	1220.0												
24				32.	16.	1270.0												
25				33.	23.	1250.0												

Individual Data Sheet 12

Note that; CM = control male; CF = control female; SM = stable vitiligo male; SF = stable vitiligo female; AM = active vitiligo male; AF = active vitiligo female; H = hormone or studied parameter.

**Prolactin** (µg/L) **(Age, Sex, & BMI)**																		
	**CM**			**CF**			**SM**			**SF**			**AM**			**AF**		
	**Age**	**BMI**	**H**	**Age**	**BMI**	**H**	**Age**	**BMI**	**H**	**Age**	**BMI**	**H**	**Age**	**BMI**	**H**	**Age**	**BMI**	**H**
**1**	18.	18.	8.0	20.	22.	20.0	23.	25.	22.0	22.	24.	35.0	21.	22.	42.0	22.	23.	80.0
**2**	18.	18.	8.0	21.	23.	20.0	21.	25.	22.0	22.	25.	35.0	21.	22.	43.0	22.	26.	80.0
**3**	19.	22.	9.0	20.	23.	21.0	23.	26.	23.0	22.	25.	33.0	21.	23.	43.0	22.	26.	90.0
**4**	19.	23.	9.0	20.	22.	21.0	35.	30.	24.0	23.	23.	32.0	22.	22.	44.0	22.	27.	90.0
**5**	18.	21.	10.0	20.	22.	22.0	35.	29.	25.0	22.	23.	32.0	21.	23.	44.0	23.	23.	91.0
**6**	40.	23.	10.0	21.	29.	22.0	35.	29.	26.0	44.	26.	30.0	34.	23.	45.0	22.	24.	92.0
**7**	40.	18.	7.0	21.	22.	23.0	45.	21.	21.0	44.	24.	30.0	34.	22.	45.0	22.	24.	95.0
**8**	41.	24.	7.0	21.	18.	23.0	45.	23.	21.0	44.	25.	36.0	34.	22.	47.0	44.	23.	75.0
**9**	41.	21.	6.0	23.	17.	25.0	46.	22.	20.0	44.	25.	36.0	35.	21.	47.0	44.	22.	76.0
**10**	42.	22.	6.0	23.	28.	25.0	44.	23.	20.0	44.	23.	37.0	46.	21.	40.0	44.	24.	77.0
**11**	34.	17.	8.5	44.	23.	19.0				36.	23.	37.0	46.	20.	40.0	43.	25.	88.0
**12**	33.	27.	7.5	44.	23.	18.0				33.	20.	38.0	45.	21.	36.0	40.	26.	89.0
**13**	33.	20.	6.6	43.	26.	19.0				33.	22.	38.0	46.	20.	42.0	35.	24.	86.0
**14**	33.	21.	8.4	43.	26.	18.0				40.	23.	35.0	46.	23.	43.0	35.	25.	85.0
**15**	32.	22.	8.3	44.	27.	17.0				29.	21.	34.0	45.	23.	43.0	33.	24.	88.0
**16**				42.	21.	16.0										32.	24.	82.0
**17**				42.	20.	17.0										31.	23.	85.0
**18**				44.	22.	22.0										34.	24.	80.0
**19**				42.	17.	21.0										33.	28.	80.0
**20**				41.	24.	20.0										32.	29.	90.0
**21**				33.	25.	25.0												
**22**				33.	26.	15.0												
**23**				31.	22.	26.0												
**24**				32.	16.	24.0												
**25**				33.	23.	20.0												

Individual Data Sheet 13

Note that; CM = control male; CF = control female; SM = stable vitiligo male; SF = stable vitiligo female; AM = active vitiligo male; AF = active vitiligo female; H = hormone or studied parameter.

**fT3**(pmol/L) **(Age, Sex, & BMI)**																		
	**CM**			**CF**			**SM**			**SF**			**AM**			**AF**		
	**Age**	**BMI**	**H**	**Age**	**BMI**	**H**	**Age**	**BMI**	**H**	**Age**	**BMI**	**H**	**Age**	**BMI**	**H**	**Age**	**BMI**	**H**
**1**	18.	18.	5.5	20.	22.	4.5	23.	25.	2.5	22.	24.	1.5	21.	22.	2.1	22.	23.	1.2
**2**	18.	18.	5.4	21.	23.	4.4	21.	25.	2.4	22.	25.	1.4	21.	22.	2.0	22.	26.	1.2
**3**	19.	22.	5.1	20.	23.	4.3	23.	26.	2.3	22.	25.	1.3	21.	23.	1.9	22.	26.	1.1
**4**	19.	23.	5.3	20.	22.	4.2	35.	30.	2.2	23.	23.	1.2	22.	22.	1.8	22.	27.	1.1
**5**	18.	21.	5.0	20.	22.	4.1	35.	29.	2.1	22.	23.	1.1	21.	23.	1.8	23.	23.	1.0
**6**	40.	23.	5.6	21.	29.	4.0	35.	29.	2.0	44.	26.	1.0	34.	23.	1.9	22.	24.	1.0
**7**	40.	18.	5.7	21.	22.	4.6	45.	21.	2.6	44.	24.	1.6	34.	22.	2.0	22.	24.	1.3
**8**	41.	24.	5.8	21.	18.	4.7	45.	23.	2.7	44.	25.	1.7	34.	22.	2.0	44.	23.	1.3
**9**	41.	21.	5.4	23.	17.	4.8	46.	22.	2.8	44.	25.	1.8	35.	21.	2.2	44.	22.	1.3
**10**	42.	22.	4.9	23.	28.	4.9	44.	23.	2.9	44.	23.	1.9	46.	21.	2.2	44.	24.	1.4
**11**	34.	17.	4.8	44.	23.	5.0				36.	23.	1.5	46.	20.	2.3	43.	25.	1.4
**12**	33.	27.	5.8	44.	23.	5.1				33.	20.	1.4	45.	21.	2.3	40.	26.	1.0
**13**	33.	20.	5.6	43.	26.	3.9				33.	22.	1.6	46.	20.	2.4	35.	24.	1.0
**14**	33.	21.	5.5	43.	26.	3.8				40.	23.	1.5	46.	23.	2.1	35.	25.	1.1
**15**	32.	22.	5.3	44.	27.	4.5				29.	21.	1.5	45.	23.	2.1	33.	24.	1.2
**16**				42.	21.	4.6										32.	24.	1.2
**17**				42.	20.	4.4										31.	23.	1.3
**18**				44.	22.	4.4										34.	24.	1.1
**19**				42.	17.	4.5										33.	28.	1.1
**20**				41.	24.	4.9										32.	29.	1.2
**21**				33.	25.	3.9												
**22**				33.	26.	4.5												
**23**				31.	22.	4.3												
**24**				32.	16.	4.6												
**25**				33.	23.	4.5												

Individual Data Sheet 14

Note that; CM = control male; CF = control female; SM = stable vitiligo male; SF = stable vitiligo female; AM = active vitiligo male; AF = active vitiligo female; H = hormone or studied parameter.

fT4 (pmol/L) **(Age, Sex, & BMI)**

	CM			CF			SM			SF			AM			AF		
	**Age**	**BMI**	**H**	**Age**	**BMI**	**H**	**Age**	**BMI**	**H**	**Age**	**BMI**	**H**	**Age**	**BMI**	**H**	**Age**	**BMI**	**H**
**1**	18.	18.	25.0	20.	22.	20.0	23.	25.	8.0	22.	24.	6.0	21.	22.	6.4	22.	23.	4.0
**2**	18.	18.	25.0	21.	23.	21.0	21.	25.	7.5	22.	25.	6.1	21.	22.	6.3	22.	26.	4.0
**3**	19.	22.	24.0	20.	23.	21.0	23.	26.	7.0	22.	25.	6.5	21.	23.	6.5	22.	26.	3.0
**4**	19.	23.	23.0	20.	22.	21.0	35.	30.	6.5	23.	23.	6.4	22.	22.	6.4	22.	27.	3.0
**5**	18.	21.	23.0	20.	22.	22.0	35.	29.	6.0	22.	23.	6.3	21.	23.	6.3	23.	23.	5.0
**6**	40.	23.	22.0	21.	29.	22.0	35.	29.	5.5	44.	26.	6.0	34.	23.	6.2	22.	24.	5.0
**7**	40.	18.	22.0	21.	22.	22.0	45.	21.	5.0	44.	24.	6.0	34.	22.	6.0	22.	24.	4.0
**8**	41.	24.	21.0	21.	18.	23.0	45.	23.	9.0	44.	25.	5.9	34.	22.	6.0	44.	23.	4.0
**9**	41.	21.	20.0	23.	17.	23.0	46.	22.	10.0	44.	25.	5.9	35.	21.	5.9	44.	22.	4.5
**10**	42.	22.	26.0	23.	28.	20.0	44.	23.	11.0	44.	23.	5.8	46.	21.	6.0	44.	24.	3.5
**11**	34.	17.	27.0	44.	23.	19.0				36.	23.	5.7	46.	20.	5.7	43.	25.	3.5
**12**	33.	27.	28.0	44.	23.	19.0				33.	20.	5.6	45.	21.	7.0	40.	26.	4.0
**13**	33.	20.	29.0	43.	26.	19.0				33.	22.	6.1	46.	20.	6.1	35.	24.	5.0
**14**	33.	21.	30.0	43.	26.	18.0				40.	23.	6.2	46.	23.	6.2	35.	25.	5.0
**15**	32.	22.	25.0	44.	27.	18.0				29.	21.	6.0	45.	23.	6.0	33.	24.	4.0
**16**				42.	21.	18.0										32.	24.	5.0
**17**				42.	20.	16.0										31.	23.	4.0
**18**				44.	22.	21.0										34.	24.	4.3
**19**				42.	17.	21.0										33.	28.	4.1
**20**				41.	24.	20.0										32.	29.	4.4
**21**				33.	25.	22.0												
**22**				33.	26.	20.0												
**23**				31.	22.	21.0												
**24**				32.	16.	23.0												
**25**				33.	23.	19.0												

Individual Data Sheet 15

Note that; CM = control male; CF = control female; SM = stable vitiligo male; SF = stable vitiligo female; AM = active vitiligo male; AF = active vitiligo female; H = hormone or studied parameter.

**TSH** (mIU/L) **(Age, Sex, & BMI)**																		
	**CM**			**CF**			**SM**			**SF**			**AM**			**AF**		
	**Age**	**BMI**	**H**	**Age**	**BMI**	**H**	**Age**	**BMI**	**H**	**Age**	**BMI**	**H**	**Age**	**BMI**	**H**	**Age**	**BMI**	**H**
**1**	18.	18.	4.0	20.	22.	4.5	23.	25.	6.0	22.	24.	7.0	21.	22.	7.0	22.	23.	8.0
**2**	18.	18.	4.1	21.	23.	4.6	21.	25.	6.1	22.	25.	7.1	21.	22.	7.3	22.	26.	8.0
**3**	19.	22.	4.1	20.	23.	4.7	23.	26.	6.1	22.	25.	7.2	21.	23.	7.4	22.	26.	8.1
**4**	19.	23.	4.2	20.	22.	4.8	35.	30.	6.2	23.	23.	7.3	22.	22.	7.5	22.	27.	8.1
**5**	18.	21.	4.2	20.	22.	4.8	35.	29.	6.2	22.	23.	7.4	21.	23.	7.4	23.	23.	8.2
**6**	40.	23.	4.3	21.	29.	4.9	35.	29.	6.3	44.	26.	7.5	34.	23.	7.4	22.	24.	8.2
**7**	40.	18.	4.5	21.	22.	4.9	45.	21.	6.4	44.	24.	6.5	34.	22.	6.8	22.	24.	8.3
**8**	41.	24.	3.9	21.	18.	5.0	45.	23.	5.5	44.	25.	6.6	34.	22.	6.6	44.	23.	8.3
**9**	41.	21.	3.9	23.	17.	5.0	46.	22.	5.5	44.	25.	6.7	35.	21.	7.0	44.	22.	8.4
**10**	42.	22.	3.8	23.	28.	4.0	44.	23.	5.4	44.	23.	6.8	46.	21.	7.0	44.	24.	8.4
**11**	34.	17.	3.8	44.	23.	4.0				36.	23.	6.9	46.	20.	7.0	43.	25.	7.5
**12**	33.	27.	3.5	44.	23.	4.1				33.	20.	7.5	45.	21.	7.2	40.	26.	7.5
**13**	33.	20.	3.4	43.	26.	4.1				33.	22.	6.5	46.	20.	6.5	35.	24.	7.6
**14**	33.	21.	4.0	43.	26.	4.2				40.	23.	6.0	46.	23.	6.8	35.	25.	7.6
**15**	32.	22.	4.0	44.	27.	4.2				29.	21.	7.0	45.	23.	7.0	33.	24.	7.7
**16**				42.	21.	4.3										32.	24.	7.7
**17**				42.	20.	4.3										31.	23.	7.8
**18**				44.	22.	4.5										34.	24.	7.8
**19**				42.	17.	4.6										33.	28.	8.0
**20**				41.	24.	4.5										32.	29.	8.0
**21**				33.	25.	4.4												
**22**				33.	26.	4.5												
**23**				31.	22.	4.7												
**24**				32.	16.	4.4												
**25**				33.	23.	4.5												
